# Cell-type-specific chromatin occupancy by the pioneer factor Zelda drives key developmental transitions in *Drosophila*

**DOI:** 10.1038/s41467-021-27506-y

**Published:** 2021-12-09

**Authors:** Elizabeth D. Larson, Hideyuki Komori, Tyler J. Gibson, Cyrina M. Ostgaard, Danielle C. Hamm, Jack M. Schnell, Cheng-Yu Lee, Melissa M. Harrison

**Affiliations:** 1 Department of Biomolecular Chemistry, University of Wisconsin School of Medicine and Public Health, Madison, WI USA; 2 Life Sciences Institute, University of Michigan, Ann Arbor, MI USA; 3 Department of Cell and Developmental Biology, University of Michigan Medical School, Ann Arbor, MI USA; 4 Division of Genetic Medicine, Department of Internal Medicine and Comprehensive Cancer Center, University of Michigan Medical School, Ann Arbor, MI USA; 5 Present Address: Human Biology Division, Fred Hutchinson Cancer Research Center, Seattle, WA USA; 6 Present Address: Department of Stem Cell and Regenerative Medicine, Keck School of Medicine of the University of Southern California, Los Angeles, CA USA

**Keywords:** Chromatin, Stem cells

## Abstract

During *Drosophila* embryogenesis, the essential pioneer factor Zelda defines hundreds of *cis-*regulatory regions and in doing so reprograms the zygotic transcriptome. While Zelda is essential later in development, it is unclear how the ability of Zelda to define *cis-*regulatory regions is shaped by cell-type-specific chromatin architecture. Asymmetric division of neural stem cells (neuroblasts) in the fly brain provide an excellent paradigm for investigating the cell-type-specific functions of this pioneer factor. We show that Zelda synergistically functions with Notch to maintain neuroblasts in an undifferentiated state. Zelda misexpression reprograms progenitor cells to neuroblasts, but this capacity is limited by transcriptional repressors critical for progenitor commitment. Zelda genomic occupancy in neuroblasts is reorganized as compared to the embryo, and this reorganization is correlated with differences in chromatin accessibility and cofactor availability. We propose that Zelda regulates essential transitions in the neuroblasts and embryo through a shared gene-regulatory network driven by cell-type-specific enhancers.

## Abstract (web summary)

The pioneer transcription factor Zelda is essential for the reprogramming of germ cells to totipotent cells of the early Drosophila embryo. Here the authors examine the function of Zelda later in development in the larval brain to show that Zelda promotes the undifferentiated stem-cell fate. They further identify factors that limit the reprogramming capacity of this pioneer factor.

## Introduction

During development, the genome is differentially interpreted to give rise to thousands of distinctive cell types. Once terminally differentiated, cells within an organism are generally incapable of transitioning to a less differentiated fate. By contrast, in cell culture the addition of a cocktail of transcription factors can reprogram differentiated cells back to a pluripotent state. Many of these reprogramming factors, including Oct4, Sox2, and Klf4, function as pioneer-transcription factors: a specialized set of transcription factors that can bind DNA within the context of nucleosomes, facilitate chromatin accessibility and subsequent binding by additional transcription factors^1–5^. These features of reprogramming pioneer factors allow them to gain access to silenced regions of the genome and drive new gene expression profiles to change cell fate. Indeed, misexpression of pioneer factors within an organism leads to dramatic gene expression changes that can cause disease^6–10^. Despite the ability of these factors to engage silenced portions of the genome, there are barriers to pioneer-factor binding and efficient reprogramming^4,11–17^. Many studies have leveraged the advantages of cell culture systems to identify impediments to pioneer-factor binding and reprogramming. However, many fewer studies have identified limitations to pioneer-factor-driven cell-fate changes within the context of an entire, developing organism.

Immediately following fertilization, the specified germ cells must be reprogrammed to form the totipotent cells that can ultimately differentiate to generate a new organism. During this time, the zygotic genome is transcriptionally silent, and maternally deposited mRNAs and proteins control early embryonic development^18–21^. This maternal-to-zygotic transition (MZT) is necessary for development and is orchestrated, in part, by factors that reprogram the zygotic genome for transcriptional activation^19,20^. Factors that activate the zygotic genome have been identified in many species and all share essential features of pioneer factors. Perhaps the best characterized of these transcriptional regulators is Zelda (Zld), which we and others have shown functions as a pioneer factor to reprogram the early embryonic genome in *Drosophila melanogaster*^22–28^.

In the early embryo, Zld binding is driven strongly by sequence with between 40–65% of the canonical Zld-binding motifs (CAGGTAG) bound by Zld during the MZT^22^. This binding is distinctive even for pioneer-transcription factors^12,29–31^. For example, the extensively studied pioneer factor FOXA2 only binds ~10% of its motifs in a variety of different cell types^12^. The chromatin environment in the early embryo is naïve as compared to later in development^32^, and this may contribute to the widespread occupancy of CAGGTAG motifs by Zld at this stage of development. In the *Drosophila* embryo, similar to other organisms, there are relatively few post-translational modifications to the histone proteins, and the genome is packaged by a unique linker histone dBigH1, which is essential for proper development^19,33,34^. In addition, early development is characterized by a series of 13, rapid, semi-synchronous nuclear divisions that each occur over approximately 10 minutes and are comprised of only synthesis (S) and division (M) phases^32^. While it is possible that the reprogramming function of Zld requires these distinctive properties of early development, Zld is also necessary for development after the MZT^26^. It remains unclear whether Zld defines *cis*-regulatory regions in tissues outside the early embryo and if so, how this activity is regulated by the cell-type-specific chromatin established during development.

**Fig. 1:**
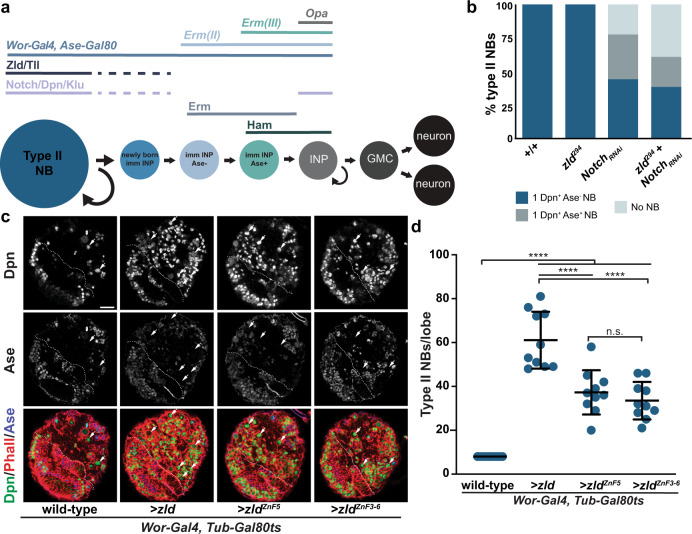
Zld promotes the undifferentiated type II neuroblast fate. **a** Expression of self-renewal factors (Notch/Dpn/Klu), Tll, Zld, Erm, and Ham and cell-type specific drivers along the type II neuroblast (NB) lineage. **b** Percent of clones of the indicated genotype with neuroblasts (NB) expressing Dpn and/or Ase or lacking cells expressing these neuroblast markers. For +/+ *n* = 14 clones, *zld*^*294*^ *n* = 16 clones, *Notch*_*RNAi*_ *n* = 9 clones, *zld*^*294*^ + *Notch*_*RNAi*_ *n* = 13 clones. **c** Immunostaining of third instar larval brain lobes without transgene expression (wild-type), expression of ectopic Zld (>*zld*), expression of Zld with a mutation in the fifth zinc finger (>*zld*^*ZnF5*^), and expression of Zld with mutations in zinc fingers 3–6 (>*zld*^*Zn3-6*^) in type I and type II neuroblasts (*Wor-Gal4, Tub-Gal80ts*) at 33 °C. White dashed line highlights the division between the optic lobe and the brain. White arrows indicate a selection of Dpn^+^, Ase^−^ type II neuroblasts. Scale bar, 20 µm. **d** Quantification of type II neuroblasts per lobe for the genotypes indicated. Wild-type (8 ± 0), >*zld* (61.1 ± 13), >*zld*^*ZnF5*^ (37.3 ± 10.1), and >*zld*^*Zn3-6*^ (33.5 ± 8.5). Mean number of type II neuroblasts is shown, and error bars show the standard deviation for a sample. For each genotype: *n* = 10 brains. Comparisons were performed with a one-way ANOVA with post-hoc Tukey’s HSD test; n.s. *p*-value = 0.7952, ****p-value ≤ 0.0001. Source data are provided as a Source Data file.

Asymmetric division of neural stem cells (neuroblasts) in the larval brain provide an excellent in vivo system for investigating temporal regulation of enhancer activity. In the larval brain lobe, there are predominantly two types of neural stem-cell populations: type I and type II^35–37^. Both types of neuroblasts undergo asymmetric division to self-renew and to generate a descendant that exits the multi-potent state and begins to differentiate. While type I neuroblasts directly contribute to neurogenesis, type II neuroblasts divide asymmetrically to self-renew and to generate a sibling cell that commits to an intermediate neural progenitor (INP) identity and functions as a transit-amplifying cell. One of the newly born neuroblast progeny, marked by the absence of Deadpan (Dpn) expression, first transitions into a non-Asense-expressing (Dpn^−^Ase^−^) immature INP, and then, after three to four hours, to an Asense-expressing (Dpn^−^Ase^+^) immature INP. Dpn is re-expressed in mature INPs (Dpn^+^Ase^*+*^) that undergo 6–8 rounds of asymmetric division to exclusively generate differentiated cell types (Fig. 1a). Thus, despite Dpn expression and the capacity to undergo a limited number of asymmetric divisions, INPs lack the functional characteristics of the type II neuroblast from which they are derived. These molecularly defined intermediate stages of INP commitment provide a powerful system to investigate the temporal control of enhancer activity as stem cells exit the undifferentiated state.

As in many stem-cell populations, Notch signaling is essential for maintaining type II neuroblasts in an undifferentiated state: loss of Notch signaling causes premature commitment of neural stem cells to INP fate, and constitutive activation leads to extra neuroblasts^38^. However, despite reactivation of Notch signaling in the mature INPs, these cells do not revert back to stem cells. Downregulation of Notch signaling in newly born INPs allows the transcriptional repressors Earmuff (Erm) and Hamlet (Ham) to become sequentially activated during INP commitment^39,40^. Erm and Ham function through histone deacetylase 3 (Hdac3) to silence expression of *tailless (tll)*, which encodes a master regulator of type II neuroblast fate^41^. Suppressor of Hairless (Su(H)), the DNA-binding partner of Notch, binds the *tll* locus in larval brain neuroblasts, suggesting that *tll* is a Notch-target^42^. Thus, in the INPs Erm- and Ham-mediated silencing of *tll* prevents reactivation of Notch signaling from inducing *tll* expression and driving their reversion to neuroblasts. Consistent with this model, INPs in *erm* or *ham*-mutant brains spontaneously revert to type II neuroblasts and knocking down Notch function can suppress the supernumerary neuroblast formation in these mutant brains^41,43^. These results support a model whereby sequential silencing by Erm and Ham during INP commitment renders the *tll* locus refractory to aberrant activation in INPs, but the precise mechanisms and the enhancers upon which these repressors act remains unclear.

Zld has been previously shown to be expressed in type II neuroblasts, but not in their progeny^44^. Thus, the type II neuroblast lineage provides a powerful system to investigate the function of this pioneer factor in a tissue apart from the early embryo. We demonstrate that Zld functions with Notch signaling to maintain type II neuroblasts in an undifferentiated state. Exogenous expression of Zld during INP commitment can promote INP reversion into neuroblasts. However, Erm and Ham expression limit this reprogramming capacity. We show that in type II neuroblasts chromatin occupancy by Zld is reorganized as compared to the embryo and that this reorganization is correlated with changes in chromatin accessibility. Nonetheless, target genes such as *dpn* and *tll* are shared between developmental stages. We propose that Zld drives key developmental transitions in the neuroblast lineage and the early embryo through a shared gene-regulatory network by binding to cell-type-specific enhancers.

## Results

### Zelda and Notch synergistically promote an undifferentiated state

Despite Zld expression being limited to the type II neuroblast, a previous study reported that knocking down *zld* function had no detectable effect on the neuroblast, but instead resulted in defects in INP proliferation^44^. This discrepancy between the Zld expression pattern and the phenotype induced by RNAi-mediated *zld* knockdown prompted us to re-evaluate the role of *zld* in the larval brain (Fig. 1a). We therefore assessed the identity of cells in mosaic clones derived from single *zld-*null mutant (*zld*^*294*^) neuroblasts using well-defined cell-fate markers. *zld*-mutant clones contained a single neuroblast and 15.4 ± 7 INPs, indistinguishable from wild-type clones (16.1 ± 6.2 INPs) (Fig. 1b, Supplementary Fig. 1a; for each genotype *n* = 10 clones). These data indicate that Zld is either dispensable in the type II neuroblast lineage or redundant pathways compensate for the absence of Zld.

Notch signaling plays an essential role in maintaining type II neuroblasts in an undifferentiated state^45–47^. We therefore tested whether Zld might act synergistically with Notch signaling to regulate type II neuroblasts. For this purpose, we assessed mosaic clones in a sensitized genetic background in which Notch function was reduced by RNAi. 22.2% of *Notch-*RNAi type II neuroblast clones lacked identifiable neuroblasts, and 33.3% of the clones contain neuroblasts with reduced cell diameter and Ase expression; two characteristics indicative of differentiation (*n* = 9 clones; Fig. 1b). Simultaneous removal of *zld* enhanced this phenotype. 38.5% of *Notch*-RNAi, *zld-*mutant clones contained no neuroblasts, and 23% of clones contained neuroblasts with markers indicative of differentiation (*n* = 13 clones; Fig. 1b). These data support a model in which Zld functions together with Notch to maintain type II neuroblasts in an undifferentiated state.

To further test if Zld promotes an undifferentiated state in type II neuroblasts, we overexpressed Zld using a series of UAS-*zld* transgenes under the control of a heat-inducible pan-neuroblast Gal4 driver (*Wor-Gal4, TubGal80ts*), allowing us to limit expression to the larval stages. While wild-type third instar larval brain lobes invariably contained 8 type II neuroblasts, 72 h of Zld overexpression starting in the L1 larval stage resulted in 61.1 ± 13 type II neuroblasts per lobe (*n* = 10 brains per genotype; Fig. 1c, d). In situ hybridization for the type II neuroblast marker *Sp1* confirmed the type II neuroblast identity of these supernumerary neuroblasts (Supplementary Fig. 1b). Zld is a zinc-finger transcription factor that binds to DNA in a sequence-specific manner that depends on a cluster of four zinc fingers in the C-terminus of the protein^25^. Mutation of either a single zinc finger or all four zinc fingers abrogates the ability of Zld to bind DNA and activate gene expression^25^. To determine if Zld overexpression drives this supernumerary type II neuroblast formation by binding DNA and regulating gene expression, we overexpressed Zld protein with either a mutation in a single zinc finger of the DNA-binding domain (ZnF^5^) or all four zinc fingers of the DNA-binding domain mutated (ZnF^3–6^). Overexpressing either protein resulted in supernumerary type II neuroblast formation (37.3 ± 10.1 and 33.5 ± 8.5 type II neuroblasts per lobe, respectively; *n* = 10 brains per genotype), but expression of either mutant protein generated significantly fewer supernumerary neuroblasts than overexpression of wild-type Zld (Fig. 1c, d). Together these data suggest that Zld promotes an undifferentiated state in type II neuroblasts and that this function is at least partially dependent on the ability of Zld to bind DNA in a sequence-specific manner.

### Zelda promotes an undifferentiated state by activating *dpn*

**Fig. 2:**
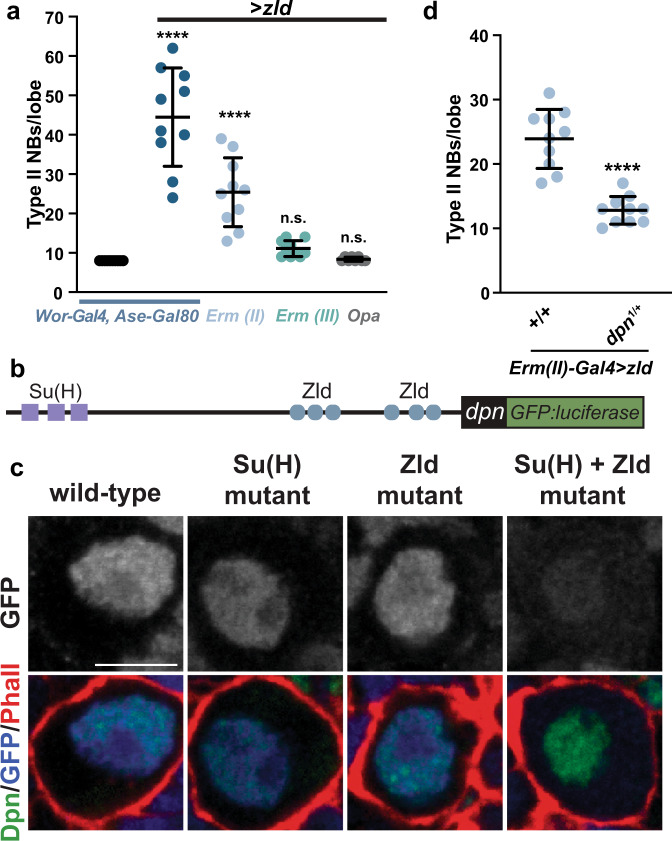
Zld promotes an undifferentiated state by activating *dpn* expression. **a** Quantification of type II neuroblasts per lobe when Zld expression is driven by the indicated drivers (see Fig. 1a for expression timing). Mean number of type II neuroblasts is shown, and error bars show the standard deviation for a sample. *Wor-Gal4, Ase-Gal80* (8 ± 0) *n* = 10 brains, *Wor-Gal4, Ase-Gal80* > *zld* (44.5 ± 12.5) *n* = 10 brains, *Erm-Gal4(II)* > *zld* (25.4 ± 8.7) *n* = 10 brains, *Erm-Gal4(III)* > *zld* (11.1 ± 2.0) *n* = 10 brains, *Opa-Gal4* > *zld* (8.4 ± 0.5) *n* = 8 brains. Significance is compared to *Wor-Gal4, Ase-Gal80* with a one-way ANOVA with post-hoc Dunnett’s multiple comparisons test; n.s. *p*-value = 0.7269 (*Erm-Gal4(III)* > *zld)*, 0.9998 (*Opa-Gal4* > *zld)*, ****p-value ≤ 0.0001. **b** Diagram of the *cis-*regulatory region of *dpn* used to create GFP:luciferase reporter constructs. Relative positions of Su(H)- and Zld-binding motifs are indicated. **c** Representative images of GFP expression (top*)* in type II neuroblasts of animals expressing transgenes containing the wild-type binding sites (*n* = 10 neuroblasts), mutated Su(H)-binding sites (*n* = 7 neuroblasts), mutated Zld-binding sites (*n* = 9 neuroblasts), or mutated Su(H)- and Zld-binding sites (*n* = 8 neuroblasts). Staining for markers of the neuroblasts are shown below. Scale bar, 5 µm. **d** Quantification of the number of type II neuroblasts per lobe upon exogenous expression of *zld* in immature INPs using the *Erm-Gal4(III)* driver in either wild-type (23.9 ± 4.6) or *dpn*^*−/+*^ (12.8 ± 2.1) larvae (*n* = 10 brains per genotype). Mean number of type II neuroblasts is shown, and error bars show the standard deviation for a sample. Comparison done using a two-tailed Student’s *t*-test with equal variance; ****p-value = 1.7565 × 10^−6^. Source data are provided as a Source Data file.

Our data showed a genetic interaction between Zld and Notch, supporting a role for Zld in promoting an undifferentiated state in type II neuroblasts by functioning in parallel to or downstream of Notch. Aberrant activation of Notch signaling in either immature or mature INPs drives supernumerary neuroblast formation^48^. If Zld acts downstream of Notch to promote the undifferentiated state, then Zld misexpression in either immature INPs or mature INPs should similarly induce supernumerary neuroblasts. To test this, we induced Zld expression in different cell types along the type II neuroblast lineage and scored for supernumerary neuroblasts in third instar larval brains. Because no Gal4 drivers are exclusively active in immature INPs, we compared the effects of Zld misexpression in both immature INPs and mature INPs (driven by *Erm-Gal4*) to expression only in INPs (driven by *Opa-Gal4)* (Fig. 1a). Zld overexpression throughout the type II neuroblast lineage driven by *Wor-Gal4, Ase-Gal80* led to 44.5 ± 12.5 type II neuroblasts per brain lobe as compared to the 8 ± 0 type II neuroblasts consistently identified in wild-type brains (*n* = 10 brains; Fig. 2a). Zld misexpression in all immature INPs driven by *Erm(II)-Gal4* resulted in 25.4 ± 8.7 type II neuroblasts per lobe (*n* = 10 brains). While fewer supernumerary type II neuroblasts (11.1 ± 2.0 type II neuroblasts per lobe; *n* = 10 brains) were identified when Zld misexpression was limited to late immature INPs and mature INPs (*Erm(III)-Gal4*), these data demonstrate that Zld misexpression can revert partially differentiated neuroblast progeny back to an undifferentiated stem-cell fate. Unlike Zld misexpression in immature INPs, expression of Zld in mature INPs driven by *Opa-Gal4* was not sufficient to induce supernumerary neuroblast formation (8.4 ± 0.5 type II neuroblasts per lobe; *n* = 8 brains). Thus, the ability of misexpression of Zld to promote the undifferentiated state is limited along the neuroblast lineage. This stands in contrast to aberrant Notch activation in mature INPs that can result in supernumerary neuroblasts. Thus, Zld promotes an undifferentiated state by functioning in parallel to, and not downstream of, Notch.

Notch functions by promoting expression of transcriptional repressors, including Dpn, to maintain type II neuroblasts in an undifferentiated state^45,46,49,50^. However, Dpn remains expressed in type II neuroblasts even in the absence of Notch signaling, suggesting that additional activators can drive expression^49^. Zld binds to the *cis*-regulatory regions of *dpn* in the early embryo, and embryonic *dpn* expression depends on maternally encoded Zld (Supplementary Fig. 2a)^22,23^. Thus, we hypothesized that Zld may promote an undifferentiated state in type II neuroblasts by functioning along with Notch to activate *dpn* expression.

To test the role of Zld in driving *dpn* expression, we generated transgenic reporters containing the *cis-*regulatory region of *dpn* driving GFP:luciferase (Fig. 2b). This five kilobase region includes regulatory regions necessary for expression in both the embryo and neuroblasts, including several Zld-bound loci, as identified by ChIP-seq in the early embryo, and a cluster of previously identified binding sites for the Notch-binding partner Su(H) (Fig. 2b)^22,49^. In addition to the wild-type reporter, we created reporters with either the Zld-binding motifs, the Su(H)-binding motifs, or both Zld and Su(H)-binding motifs mutated. Similar to the endogenous locus, expression of the reporter depended on Zld binding for expression in the embryo^22,23^ (Supplementary Fig. 2b). GFP expression was evident in type II neuroblasts of larva carrying the reporter with the wild-type *dpn*-regulatory region (Fig. 2c; Supplementary Fig. 2c). Mutation of either Su(H) or Zld-binding motifs reduced, but did not eliminate, GFP expression. Only mutation of both sets of binding motifs abrogated expression, demonstrating that *dpn* is a target of Zld in type II neuroblasts, similar to the embryo, and that Zld functions redundantly with Notch signaling to activate *dpn* expression.

Because of the ability of Zld and Notch to both activate *dpn* expression, to determine the functional significance of Zld-mediated activation of *dpn* expression we needed to test the effect of Zld expression in the absence of active Notch signaling. For this purpose, we focused on misexpression of Zld in immature INPs where Notch signaling is not active (Fig. 1a). The supernumerary phenotype caused by Zld misexpression in immature INPs driven by *Erm(II)-Gal4* (23.9 ± 4.6 type II neuroblasts per lobe; *n* = 10 brains) was strongly suppressed by loss of a single copy of *dpn* (12.8 ± 2.1 type ll neuroblasts per lobe; *n* = 10 brains; Fig. 2d). These data demonstrate that Zld expression in immature INPs promotes reversion to an undifferentiated stem-cell fate at least in part by driving *dpn* expression. We note that while *Erm(II)-Gal4* is active in both immature INPs and mature INPs, this observed suppression is not due to reducing Zld-induced *dpn* expression in mature INPs, where Notch signaling is reactivated. This is because *dpn* overexpression in mature INPs cannot induce a supernumerary type II neuroblast phenotype. Thus, Zld functions in parallel to Notch to maintain type II neuroblasts in an undifferentiated state by activating *dpn* expression, and misexpression of Zld in the partially differentiated neuroblast progeny can revert them to an undifferentiated state by activating *dpn* expression.

### Zelda binds thousands of sites in type II neuroblasts

**Fig. 3:**
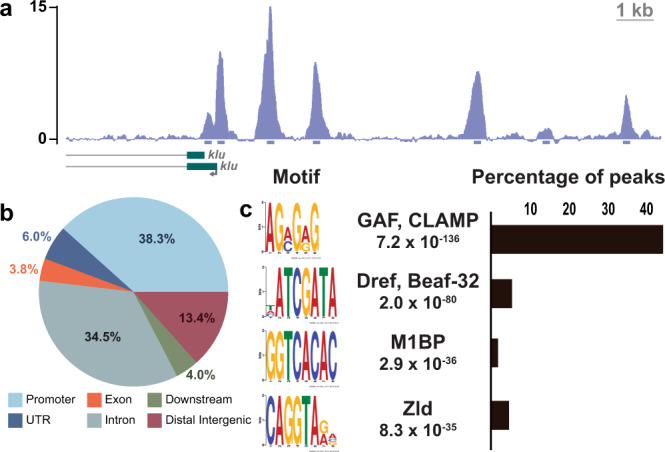
Zld binds to thousands of loci in type II neuroblasts. **a** Representative genome browser tracks at the *klu* locus of Zld ChIP-seq from *brat*^*11/Df(2L)Excel8040*^ brains. 200 bp regions surrounding the peak summits are shown below the track. **b** Pie chart of the genomic distribution of Zld-binding sites in type II neuroblasts. Promoters are defined as −500 to +150 bp from the transcription start site. **c** List of enriched motifs for Zld-bound regions in type II neuroblasts identified by MEME-suite (*left*). The significance value is based on the log likelihood ratio, width, sites, background, and size of the input sequence. Histogram of the fraction of the 12,208 peaks identified for Zld ChIP-seq containing the GAF, CLAMP (ACMGRG), Dref, Beaf-32 (HATCGATA), M1BP (GGTCACA), or Zld (CAGGTARV) motif (*right)*.

**Fig. 4:**
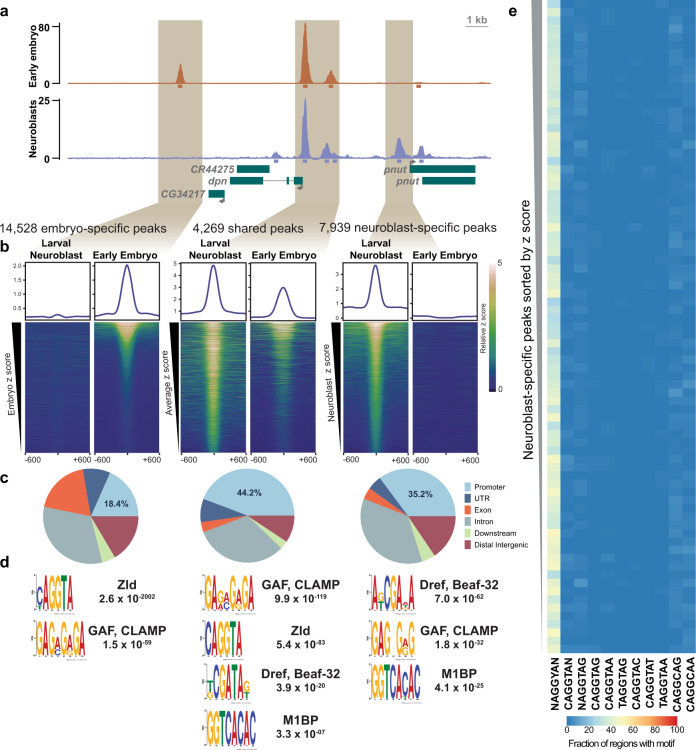
Zld occupancy in type II neuroblasts is driven by a feature apart from DNA sequence. **a** Representative genome browser tracks for Zld ChIP-seq from stage 5 embryos^22^ and *brat*^*11/Df(2L)Excel8040*^ brains centered on the *dpn* locus. 200 bp regions surrounding the peak summits are shown below the tracks. **b** Heatmaps centered on the ChIP peak summit with 600 bp flanking sequence for the embryo-specific peaks ranked by embryo-specific peak signal, shared peaks ranked by the average signal across the embryo and type II neuroblasts, and type II neuroblast-specific peaks ranked by type II neuroblast-specific peak signal. Colors indicate relative ChIP-seq z score. Average z score profile is shown above each heat map. **c** Pie charts of the genomic distribution of Zld-binding sites in each peak class. Promoters are defined as −500 to +150 bp from the transcription start site. **d** Motif enrichment for each peak class as determined by MEME-suite. The significance value is based on the log likelihood ratio, width, sites, background, and size of the input sequence. **e** Enrichment of Zld-binding motif variants among type II neuroblast-specific Zld peaks. Peaks are sorted based on z score from highest (top) to lowest (bottom) and binned into groups of 100. Color of each cell represents the percent of peaks within each bin containing the indicated motif.

Having demonstrated that Zld promotes the undifferentiated type II neuroblast fate and that this activity is dependent, at least partially, on DNA binding, we used chromatin immunoprecipitation coupled with high-throughput sequencing (ChIP-seq) to identify Zld-binding sites in the type II neuroblasts. These experiments required enriching for type II neuroblasts, as wild-type larval brains only contain eight type II neuroblasts per lobe. For this purpose, we performed ChIP-seq on third instar larval brains dissected from larvae that are mutant for *brain tumor* (*brat*^*11/Df(2L)Exel8040*^). These *brat*-mutant brains contain thousands of type II neuroblasts at the expense of other cell types and are a well characterized model for studying the transition from an undifferentiated stem-cell state to a committed INP identity^44,50,51^. Supporting the relevance of this tissue for these assays, we demonstrated that Zld levels within a single neuroblast are equivalent between wild-type and *brat*-mutant brains (Supplementary Fig. 3a). Furthermore, these brains provide a biologically relevant system as the supernumerary neuroblasts are capable of differentiating along the type II lineage when the activity of genes that maintain neuroblasts in an undifferentiated state are inhibited^41^. Using the same Zld antibody previously used for Zld ChIP-seq in the early embryo^22^, ChIP-seq was performed in duplicate. The high correlation between replicates (Pearson’s correlation = 0.89) (Supplementary Fig. 3b), allowed us to identify 12,208 high-confidence peaks. Among the Zld-bound regions were the regulatory regions for genes known to maintain type II neuroblasts in an undifferentiated state, like *klumpfuss* (*klu)*^47,52^ (Fig. 3a). Identified Zld-binding sites were located in promoters and enhancers and were enriched for the known Zld-binding motif, CAGGTA (Fig. 3b, c). Supporting the functional relevance of these Zld-binding sites, we identified robust peaks in the *dpn cis*-regulatory region that correspond to those regions mutated in our transgenic assays (Fig. 4a). De novo motif enrichment also identified motifs known to be bound by additional proteins that have important functions in promoters as well as in three-dimensional chromatin organization^53–56^ (Fig. 3c).

### Zelda binds distinct sites in the type II neuroblasts and early embryo

In the early embryo, Zld binding is distinctive from that of other transcription factors. Zld binding is driven by DNA sequence with 64% of the canonical Zld-binding motifs (CAGGTAG) bound by Zld in the very early embryo^22^. Zld is a pioneer factor that can bind to nucleosomal DNA^57^, and this capacity likely contributes to the unique binding profile in the early embryo. However, the naïve chromatin environment of the early embryo may also contribute to the unique binding profile of Zld at this stage. To determine the relative contributions of the pioneering activity of Zld and the naïve chromatin environment to the binding profile of Zld, we compared the binding of Zld in the type II neuroblasts to that of the early embryo. We realigned previously published ChIP-seq data for Zld from stage 5 embryos^22^ to the dm6 genome release, using the same parameters for aligning, filtering and peak calling as was used for the neuroblast ChIP-seq data (Supplementary Data 1). This allowed us to compare Zld-bound regions between the embryo and the larval type II neuroblasts. As might be predicted for a pioneer factor, we identified 4,269 regions that were bound by Zld at both stages of development, including the *dpn cis*-regulatory region (Fig. 4a, b). Contrary to our expectations for a pioneer factor whose binding is driven strongly by sequence, we identified many more regions that were uniquely bound at a single stage of development (14,528 regions specifically bound in the stage 5 embryo and 7,939 regions specifically bound in the type II neuroblasts) (Fig. 4b). These uniquely bound regions were not the result of differences in ChIP efficiency between the tissues as these regions include some of those with the highest relative peak height (Fig. 4b). Because Zld binding in the embryo was strongly driven by sequence, the unique binding profile identified in the type II neuroblasts was unexpected. We therefore sought to confirm the identified Zld-binding sites using an additional antibody. For this purpose, we verified expression of our previously engineered endogenously sfGFP-tagged Zld in the larval neuroblasts (Supplementary Fig. 3c)^24^ and used an anti-GFP antibody for ChIP-seq to identify binding of sfGFP-Zld in *brat*-mutant brains. These experiments confirmed the cell-type-specific Zld-binding profile (Supplementary Fig. 3d–g).

To understand what features shape Zld binding, we determined the genomic distribution of Zld-bound regions specific to the embryo, specific to the type II neuroblasts, and those shared between both cell types (Fig. 4c). Regions bound by Zld in the type II neuroblasts, both those shared with the embryo and those unique to the neuroblasts, were enriched for promoters (44.2% and 35.2%, respectively; Fig. 4c) as compared to regions bound by Zld solely in the embryo (18.4%; *p*-value < 2.2e-16) or randomized regions of the genome (10.4%; *p*-value < 2.2e-16) (Fig. 4c, Supplementary Fig. 4a). Indeed, de novo motif analysis of Zld-bound regions identified multiple promoter-enriched sequence elements in these regions (Fig. 4d). Similar to previous analysis of Zld-binding sites in the embryo, Zld-bound regions unique to the embryo were strongly enriched for the canonical Zld motif^22^ (Fig. 4d). Unexpectedly, Zld-bound regions unique to the type II neuroblasts were not enriched for the Zld-binding motif (Fig. 4d). Instead, motifs of known promoter-binding factors (Dref/Beaf-32, GAF/CLAMP, and M1BP) were enriched (Fig. 4d, Supplementary Fig. 4b). These factors have insulator function and bind in the promoters of housekeeping and constitutively active genes, where a large portion of chromatin boundaries are located^53–56^. Indeed, when promoters were removed from the set of Zld-bound regions unique to the type II neuroblasts, de novo motif analysis did not enrich for these sequences, suggesting that the enrichment was due to Zld binding to promoters. Because bulk analysis can obscure sequences that might be enriched in a subset of bound regions, we binned Zld-bound regions by peak height and identified the fraction of regions in each bin that was enriched for the canonical Zld-binding motif or variants of this motif (Fig. 4e, Supplementary Fig. 4c–f). As had been previously reported, Zld-bound regions in the embryo are highly enriched for the Zld-binding motif. By contrast, Zld-bound regions in the type II neuroblasts are only weakly enriched for a degenerate version of the canonical CAGGTA Zld-binding motif (Fig. 4e). Together this analysis supports a model whereby in the embryo Zld binding is driven largely by sequence, but in the type II neuroblasts genomic features apart from sequence shape Zld binding.

### Chromatin accessibility correlates with Zelda binding and function

**Fig. 5:**
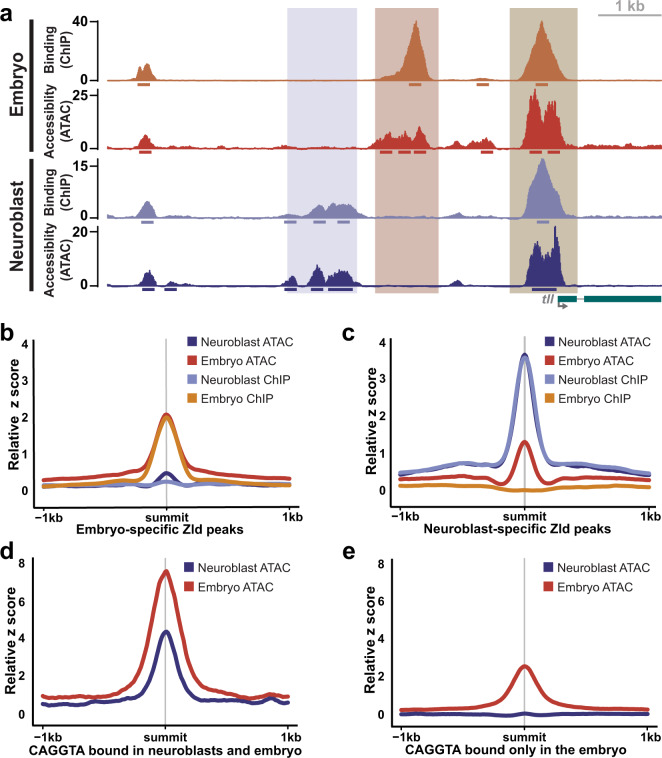
Cell-type-specific differences in chromatin accessibility correlate with cell-type-specific Zld binding. **a** Genome browser tracks of Zld binding (ChIP-seq) and chromatin accessibility (ATAC-seq) in stage 5 embryos and type II neuroblasts of *brat*^*11/Df(2L)Excel8040*^ brains at the *tll* locus. Peak regions are indicated below the tracks. Highlighted areas indicate regions bound by Zld and accessible only in neuroblasts (blue), only in embryos (orange) and in both (brown). **b, c** z score for ChIP-seq and ATAC-seq centered on Zld ChIP-seq peaks specific to the embryo (**b**) or Zld ChIP-seq peaks specific to the type II neuroblasts (**c**). **d, e** z score for ATAC-seq from the embryo and type II neuroblasts centered on the shared Zld ChIP-seq peaks containing a CAGGTA motif (**d**) or Zld ChIP-seq peaks specific to the embryo containing a CAGGTA motif (**e**).

Because chromatin accessibility is known to influence the access of transcription factors to the underlying DNA, we assayed chromatin accessibility in brains enriched for type II neuroblasts using the Assay for Transposase-Accessible Chromatin using sequencing (ATAC-seq)^58^ (Supplementary Fig. 5a). We identified 58,551 accessible regions in the type II neuroblasts, including promoters of genes encoding factors that promote the maintenance of the undifferentiated state such as Dpn, Klu and Enhancer of split mγ (E(spl)mγ) (Supplementary Fig. 5b). Ninety two percent of loci bound by Zld in type II neuroblasts overlapped with regions of accessible chromatin as assayed by ATAC-seq, including both promoters and upstream *cis-*regulatory modules (Fig. 5a). This correlation between Zld binding and open chromatin was similar to the previously reported association between Zld-bound regions and chromatin accessibility in the early embryo^22^. To better understand this relationship between accessibility and Zld binding, we used recently published ATAC-seq data generated in stage 5 embryos^59^ to allow us to compare accessibility and Zld binding in both the embryo and type II neuroblasts. This analysis showed that while Zld-bound loci and regions of open chromatin differ between the two cell types, there is a correlation between binding and accessibility in both (Fig. 5a, Supplementary Fig. 5c). Regions bound by Zld only in the embryo are more accessible in the embryo than in the type II neuroblasts (Fig. 5b). Similarly, regions bound specifically in the type II neuroblasts are more accessible in the neuroblasts than the embryo (Fig. 5c).

At a subset of Zld-bound regions in the early embryo, Zld is required for chromatin accessibility^23,60^. Thus, the correlation between Zld binding and open chromatin might be due to Zld-mediated chromatin accessibility. However, chromatin accessibility also influences transcription-factor binding, such that accessibility may guide Zld binding to some loci. Therefore, we analyzed chromatin accessibility at regions containing the canonical Zld-binding CAGGTA motif. CAGGTA-containing regions bound in the type II neuroblasts and embryo are accessible in both cell types (Fig. 5d). By contrast, CAGGTA-containing regions that are only bound in the embryo are only accessible in the embryo (Fig. 5e). Thus, in the type II neuroblasts Zld does not bind to regions containing the canonical Zld motif if the region is not accessible and suggests that the correlation between Zld binding and open chromatin in the neuroblasts may result from Zld occupying regions of accessible chromatin rather than Zld binding driving the accessibility.

### Zelda-binding sites identify cell-type-specific enhancers

**Fig. 6:**
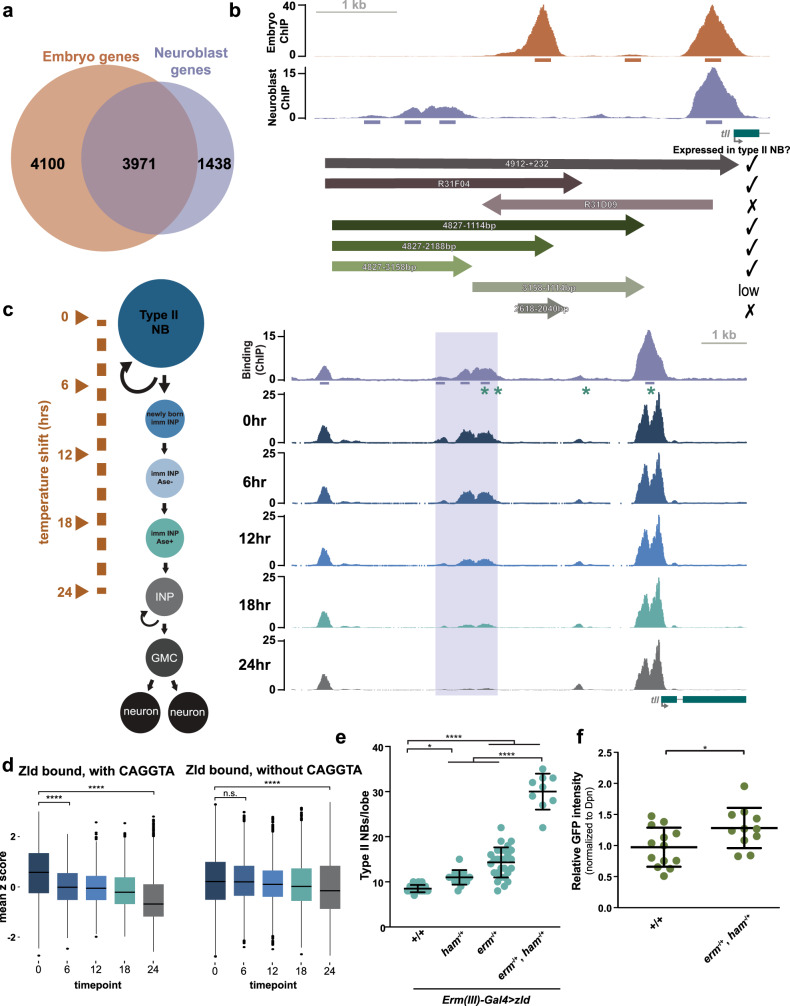
Erm and Ham limit the ability of Zld to activate a type II neuroblast-specific *tll* enhancer. **a** Venn diagram of the genes associated with Zld ChIP-seq peaks from the early embryo and type II neuroblasts. **b** Genome browser tracks of Zld binding (ChIP-seq) in stage 5 embryos and type II neuroblasts at the *tll* locus with arrows below indicating genomic regions tested for GFP expression in type II neuroblasts. Arrows show the relative direction of incorporation into transgenic reporters, and numbers indicate the sequence included relative to the transcription start site or the FlyLight clone number. **c** Genome browser tracks of Zld ChIP-seq in type II neuroblasts of *brat*^*11/Df(2L)Excel8040*^ brains and of ATAC-seq of the *tll* locus from *brat*^*11/Df(2L)Excel8040*^ brains at the indicated time points following a temperature shift that initiates synchronous differentiation. Diagram to the left indicates approximate cell types enriched at each timepoint. 200 bp regions surrounding the ChIP peak summit and Erm-binding motifs (AAAWGVVCMNH) are shown below the top track by an asterisk. **d** z score of ATAC-seq signal at 0, 6, 12, 18, and 24 h after induced differentiation at regions that are bound by Zld in the neuroblasts and contain a CAGGTA motif (left) or do not contain a CAGGTA motif (right). The horizontal line indicates the median z score, the lower and upper limits of the box correspond to the first and third quartiles, the upper whisker extends from the box to the largest value (no larger than 1.5 times the distance between first and third quartiles), the lower whisker extends from the box to the smallest value (no smaller than 1.5 times the distance between first and third quartiles) and data beyond the whiskers are outlying points. Comparisons were performed with a two-sided Wilcoxon rank sum test; n.s. *p*-value = 0.84, ****p-value ≤ 0.0001. **e** Quantification of type II neuroblasts per lobe upon Zld expression in immature INPs (*Erm-Gal4(III))* in wild-type (8.5 ± 0.8) *n* = 23 brains, *ham*^*−/+*^ (11 ± 1.6) *n* = 15 brains, *erm*^*−/+*^ (14.3 ± 3.3) *n* = 28 brains, *erm*^*−/+*^*,ham*^*−/+*^ (30 ± 4.0) *n* = 9 brains. Mean number of type II neuroblasts is shown, and error bars show the standard deviation for a sample. Comparisons were performed with a one-way ANOVA with post-hoc Tukey’s HSD test; *p-value = 0.0265, ****p-value ≤ 0.0001. **f** Quantification of GFP intensity relative to Dpn in type II neuroblasts of wild-type (*n* = 13 neuroblasts) and *erm, ham* double heterozygotes (*n* = 11 neuroblasts) in which GFP is driven by the neuroblast-specific enhancer (4827-3158 downstream of transcription start site). Mean relative GFP intensity is shown, and error bars show the standard deviation for a sample. Comparisons were performed by an unpaired two-tailed Student’s *t*-test. *p-value = 0.0272. Source data are provided as a Source Data file.

Our ChIP-seq analysis revealed thousands of Zld-binding sites that were unique to either the embryo or the type II neuroblasts (Fig. 4a, b). By contrast, the majority of genes associated with Zld-bound regions in the type II neuroblasts were also associated with Zld binding in the embryo (Fig. 6a, Supplementary Data 2). This suggests that the transcriptional network regulated by Zld is partially shared between cell types. However, the identification of Zld-bound regions in neuroblasts lacking the canonical Zld-binding motif indicated that cell-type-specific constraints influence target recognition by Zld in non-embryonic cells and that the regulation of the shared gene network may depend on Zld binding to cell-type-specific genomic locations.

To begin characterizing changes that allow Zld binding to non-canonical sites, we focused on the gene *tll*, which is expressed in both the type II neuroblasts and the embryo^41,61,62^. Zld was bound to the *tll* promoter in both the embryo and type II neuroblasts, but Zld occupied different upstream regions at each developmental timepoint (Fig. 5a). To determine if these upstream regions drive cell-type-specific gene expression, we analyzed reporter expression controlled by five kilobase of *tll* upstream-regulatory sequence, containing both the neuroblast-specific and embryo-specific Zld-bound regions. In embryos, this sequence can drive expression identical to the endogenous *tll* locus, and we showed it is sufficient to drive reporter expression in type II neuroblasts mimicking endogenous Tll expression (Fig. 6b, Supplementary Fig. 6a)^62^. Prior studies demonstrated that in the early embryo a transgene containing only three kilobase of upstream-regulatory sequence (and therefore lacking the neuroblast-specific Zld-binding sites), drives expression in a pattern identical to wild-type, but with reduced levels in the posterior^62^. Thus, the region bound by Zld specifically in the type II neuroblast is not necessary for expression in the early embryo, suggesting it may define a neuroblast-specific *cis*-regulatory module.

We more specifically tested if the type II non-canonical, neuroblast-specific Zld-binding sites were required to drive expression in the neuroblasts by examining expression of transgenes from the FlyLight collection containing portions of the *tll* regulatory region. R31F04, which contains sequence corresponding to the region extending from −4.9 to −1.8 kilobase upstream of the *tll* transcriptional start site, drove expression in the type II neuroblasts (Fig. 6b). By contrast, a portion of the regulatory region that includes the embryo-specific Zld-bound region, but not the type II neuroblast-specific region (FlyLight R31D09) failed to drive expression in the type II neuroblasts. This construct is inserted in the reverse orientation compared to the endogenous locus, and, while enhancers generally function regardless of genomic orientation, it remained possible that this difference in orientation is what results in the lack of expression.

We were unable to identify and mutate the sequence that was necessary for Zld binding, as was done for the *dpn-*regulatory region, because the region underlying the type II neuroblast-specific Zld-binding sites does not contain the canonical Zld-binding motif. To test the ability of the predicted neuroblast-specific enhancer to drive expression in type II neuroblasts, we therefore created three transgenes that contained sequentially truncated portions of the *tll* upstream-regulatory sequence but included the predicted neuroblast enhancer. We used these to drive GFP expression from the *Drosophila* synthetic core promoter element (DSCP) and assayed for GFP in the type II neuroblasts (Fig. 6b). All three fragments tested were sufficient to drive reporter expression in type II neuroblasts (Fig. 6b, Supplementary Fig. 6a). To test the necessity of the predicted neuroblast-specific Zld-bound enhancer, we generated two reporters that lack this region (Fig. 6b). One reporter contained only the embryo-specific enhancer and the DSCP promoter. The other contained the embryo enhancer as well as additional sequence. The embryo-specific enhancer did not drive GFP expression in type II neuroblasts while the larger region drove only weak GFP expression (Fig. 6b, Supplementary Fig. 6b, c). Together these assays show that type II neuroblast- and embryo-specific binding by Zld identifies enhancers required for *tll* expression specifically in each cell type.

In addition to having cell-type-specific Zld occupancy, these upstream regions also show cell-type-specific patterns of chromatin accessibility (Fig. 5a) as might be predicted for cell-type-specific enhancers. In the type II neuroblast lineage, *tll* is expressed exclusively in the neuroblast and not in other cell types. Indeed, misexpression of *tll* in mature INPs results in their reversion to neuroblasts^41,61^. To investigate whether chromatin accessibility at the Zld-bound region reflects this expression pattern, we took advantage of a synchronous, time-release differentiation system we developed that recapitulates many of the gene expression changes that occur as type II neuroblasts differentiate into INPs^41^ and performed ATAC-seq at four time points spanning the first 24 h following temperature shift (approximating differentiation from type II neuroblast to INP). Consistent with *tll* expression rapidly diminishing in immature INPs, the region with the most dramatic loss in accessibility over this time course was the Zld-bound, type II neuroblast-specific *tll* enhancer (Fig. 6c).

Like *tll*, *Six4* expression significantly decreased in our synchronous, time-release differentiation system^41^. *Six4* encodes a homeodomain-containing transcription factor with known roles mesoderm specification^63,64^. Using GFP reporter expression, we confirmed that *Six4* is expressed in type II neuroblasts and not in INPs (Supplementary Fig. 7a)^65^. We further identified a neuroblast-specific Zld-bound region upstream of *Six4* that significantly lost chromatin accessibility upon neuroblast differentiation in our time-release system (Supplementary Fig. 7b). Together these data support a model in which binding of the pioneer factor Zld in neuroblasts identifies enhancers that drive type II neuroblast-specific expression of *tll* and *Six4*, and these enhancers progressively lose accessibility as expression is silenced and cells differentiate.

To determine patterns of chromatin accessibility changes during neuroblast differentiation, we performed k-means clustering on all regions that change in accessibility during our ATAC-seq time course. We identified six clusters with distinct patterns of accessibility over the time course (Supplementary Fig. 5d; Supplementary Data 3). Clusters 4, 5, and 6 were defined by regions that decreased in accessibility over the time course and were enriched for Zld-bound sites in the neuroblasts (29%) as compared to clusters that increased in accessibility over the time course (15% in Clusters 1, 2, and 3) (*p*-value < 2.2 × 10^−16^, Fishers Exact Test). To identify factors that could be driving the decrease in accessibility in these clusters, we performed motif searches at the Zld-bound sites and identified cluster 6 as having the largest percentage of sites containing the canonical Zld-binding motif (Supplementary Fig. 5e). This cluster is characterized by a rapid decrease in accessibility immediately following heat shock, corresponding to cells exiting the neuroblast fate. Because Zld levels rapidly decrease during this cellular transition, we hypothesized that these regions bound by Zld in the neuroblasts that contain the canonical Zld motif may be sites where Zld is required for accessibility. We therefore analyzed changes in chromatin accessibility during differentiation at Zld-bound sites that either had the canonical Zld-binding motif (CAGGTA) or did not. Zld-bound regions with a canonical motif rapidly lost accessibility in the first 6 h following heat shock, whereas those that lacked a canonical-binding motif more gradually lost accessibility (Fig. 6d). This suggests that at sites with the Zld motif, Zld may be responsible for promoting accessibility. By contrast, Zld-bound regions that do not contain this motif lose accessibility less rapidly following stem-cell exit and may require additional factors for this loss in accessibility.

We have recently shown that Erm and Ham function through Hdac3 to silence *tll* expression during INP commitment, and thus are factors that might be required for the more gradual loss in accessibility at some regions^41^. The timing of Erm- and Ham-mediated silencing of *tll* coincides with the loss of chromatin accessibility at the Zld-bound, type II neuroblast enhancer (Fig. 1a). This enhancer is in cluster 5, and there are Erm-binding motifs located in the enhancer region (Fig. 6c). Thus, it is possible that the chromatin changes implemented by Erm and Ham decrease chromatin accessibility and limit the ability of Zld to bind this enhancer. If misexpression of Zld in INPs is sufficient to drive Tll expression, then Zld misexpression would be expected to mimic Tll misexpression in these cells and promote supernumerary type II neuroblast formation^41,61^. However, Zld expression in INPs (driven by *Opa-Gal4)* failed to induce supernumerary type II neuroblast formation, suggesting cell-type-specific features limit the ability of Zld to activate *tll* expression (Fig. 2a). To determine if the sequential silencing of *tll* by Erm and Ham limits the ability of Zld to induce INP reversion to type II neuroblasts, we drove Zld expression in immature INPs and mature INPs (*Erm(III)-Gal4*) of brains heterozygous for null mutations in either *erm* or *ham*. While loss of single copies of either *erm* or *ham* does not result in supernumerary type II neuroblasts^41^, loss of a single copy of either of these genes enhanced the weak supernumerary phenotype caused by misexpression of Zld (14.3 ± 3.3 type II neuroblasts; *n* = 28 brains and 11 ± 1.6 type II neuroblasts; *n* = 15 brains, respectively). This effect was enhanced when copies of both *erm* and *ham* were removed (30 ± 4.0 type II neuroblasts; *n* = 9 brains) (Fig. 6e). To more directly test the role of Erm and Ham-mediated silencing on the identified *tll* neuroblast enhancer, we analyzed GFP expression driven by the neuroblast-specific *tll* enhancer in larva heterozygous for null mutants in both *erm* and *ham*. GFP expression is increased in this double heterozygous background (Fig. 6f). Together these data support a model in which type II neuroblast-specific binding of the pioneer factor Zld promotes expression of a master regulator of type II neuroblast functional identity, Tll, and that this Zld-bound enhancer is progressively silenced by Erm and Ham to inhibit reactivation in the INP. Furthermore, we propose that other neuroblast-specific Zld-binding sites, such as those upstream of *Six4*, may identify additional enhancers driving stem-cell fate in type II neuroblasts.

## Discussion

Our results demonstrate that Zld, an essential transcriptional activator of the zygotic genome, promotes the undifferentiated state in the neural stem-cell lineage of the larva and can revert partially differentiated cells to a stem cell. Other pioneer factors are known to have similar functions when misexpressed, and this can lead to disease. For example, expression of DUX4, an activator of the zygotic genome in humans, in muscle cells leads to Facioscapulohumeral muscular dystrophy (FSHD), and OCT4 and Nanog are overexpressed in undifferentiated tumors and their expression is associated with poor clinical outcomes^10,66–71^. Despite the ability of Zld to promote the undifferentiated stem-cell fate, this capacity is limited as cells differentiate to INPs. We showed that the ability of Zld to drive gene expression is limited by the repressors Erm and Ham. Because of the ability of pioneer factor misexpression to cause disease, understanding these cell-type-specific constraints on pioneer factors has important implications for our understanding of development and disease.

**Fig. 7:**
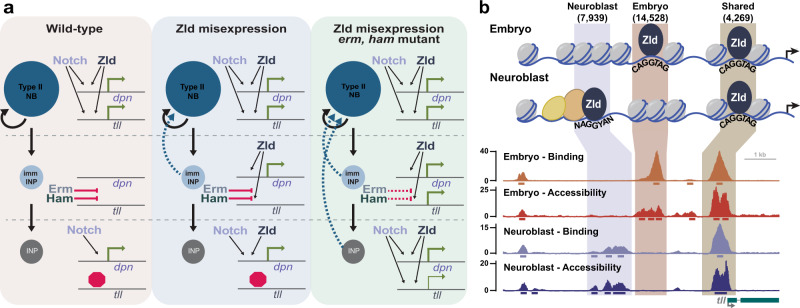
Zld promotes the undifferentiated state through neuroblast-specific enhancers that become progressively silenced during differentiation. **a** Zld functions in parallel to Notch to maintain type II neuroblasts in an undifferentiated state through activation of *tll* and *dpn*. Downregulation of Notch signaling in newly born INPs allows Erm and Ham to become sequentially activated during INP commitment. In the INPs, Erm- and Ham-mediated silencing of *tll* prevents reactivation of Notch signaling from inducing *tll* expression and driving reversion to neuroblasts. When misexpressed in immature INPs, Zld activates *dpn* expression and promotes reversion to a neuroblast. The red octagon/stop signs indicate the changes to the chromatin structure mediated by Erm and Ham that limit the ability of Zld to drive *tll* expression and therefore reprogram mature INPs. **b** Cell-type-specific binding by Zld correlates with chromatin accessibility and identifies tissue-specific enhancers. Type II neuroblast-specific Zld-binding sites are not enriched for the canonical Zld-binding motif and are instead enriched for sequences corresponding to additional co-factors that may stabilize Zld binding or promote chromatin accessibility at these loci.

Zld expression promotes the reversion of partially differentiated immature INPs to a stem-cell fate, resulting in supernumerary type II neuroblasts. Furthermore, failure to down-regulate *zld* in the newly generated INPs results in supernumerary type II neuroblasts^44^. Thus, Zld levels must be precisely controlled to allow differentiation following asymmetric division of the type II neuroblasts. Zld promotes the undifferentiated state, at least in part, through the ability to drive expression of Dpn, a key transcription factor for driving type II neuroblast self-renewal^42,45,46,49,72^. *dpn* is a target of the Notch pathway in type II neuroblasts and constitutively activated Notch signaling drives *dpn* expression^42,49^. However, loss of Notch signaling does not completely abrogate expression of known target genes, including *dpn*, suggesting that additional activators can drive expression in the absence of Notch^42,46,49^. Indeed, we show that Zld functions as such a factor in driving *dpn* expression, and loss of a single copy of *dpn* can suppress the ability of Zld to promote the reversion of immature INPs to type II neuroblasts. We propose that this redundancy with Notch is not limited to regulating *dpn* expression and that Zld and Notch may function together to regulate a number of genes required for type II neuroblast maintenance. Supporting this, Zld is bound to 49% of the identified direct Notch-target genes in neuroblasts (Supplementary Data 2)^42^. Although, Zld is not required for type II neuroblast maintenance, loss of Zld can enhance knockdown of the Notch pathway demonstrating a partially redundant requirement for these two pathways in maintaining type II neuroblast fate. Based on these data, we propose that Zld and Notch function in parallel to drive gene expression, and this redundancy robustly maintains the type II neuroblast pool (Fig. 7a).

Zld binding in the early embryo is distinctive as it is driven primarily by DNA sequence with a majority of the canonical-binding motifs occupied. This is in contrast to most other transcription factors, whose binding is influenced widely by chromatin accessibility and therefore bind only a small fraction of their canonical motifs^73–75^. Here we report the genome-wide occupancy of Zld in a tissue apart from the early embryo and begin to identify important functions for zygotically expressed Zld in a stem-cell population. While we identified thousands of loci that are occupied by Zld both in the embryo and in the larval type II neuroblasts, thousands more were unique to each cell type. This is in contrast to what we have shown for another pioneer-transcription factor, Grainy head (Grh), which has similar genomic occupancy in the embryo and larval imaginal discs^59,76^. Thus, unlike for Grh, Zld binding is cell-type-specific and likely governed by changes to the chromatin structure along with the expression of cell-type-specific transcription factors (Fig. 7b).

Despite their ability to engage nucleosomal DNA, experiments, largely in cell culture, have demonstrated that most pioneer-transcription factors show cell-type-specific chromatin occupancy^11–13,16,77^. Both chromatin state and co-factors influence the binding of pioneer factors, like Oct4^3,4,13,77^. While most pioneer factors have been studied through misexpression in culture, our data show that binding of an endogenously expressed pioneer factor within a developing organism is also cell-type specific. By analyzing genome occupancy and chromatin accessibility in two different cell types, the embryo and type II neuroblast lineage, we demonstrate that binding is highly correlated with accessibility in both cell types. While it is not possible to determine whether chromatin accessibility regulates Zld binding or Zld binding drives accessibility, we propose that at sites with the Zld motif, Zld may be responsible for promoting accessibility. While at the majority of sites bound by Zld in the larval type II neuroblasts, accessibility influences Zld occupancy. In the early embryo, Zld binds when the chromatin is naïve with relatively few chromatin marks and is rapidly replicated^32^. This Zld binding is driven largely by DNA sequence and is required for chromatin accessibility at a subset of sites^22,23,28^. Thus, in the early embryo, Zld binding can influence accessibility. However, Zld occupancy is reorganized in the type II neuroblasts such that only a fraction of the canonical-binding motifs is occupied, and those motifs that are not bound by Zld are not accessible. The small percentage of Zld-bound sites that contain the canonical Zld-binding motif in the neuroblasts rapidly loose accessibility following induced differentiation and sites that lack a canonical-binding motif more gradually loose accessibility. This suggests that at sites with the Zld motif, Zld may be responsible for promoting accessibility. In contrast to this small subset of sites, there is a significant enrichment of type II neuroblast-specific binding sites at promoters, which are known to be generally accessible in a broad range of cell types. This suggests that Zld occupancy in the type II neuroblasts is likely shaped by chromatin accessibility. Together, these data support a model whereby in the early embryo Zld can bind broadly to the naïve genome while in the neuroblasts Zld binding is limited by chromatin state. Future studies will enable the identification of what limits Zld binding and will allow for the definition of chromatin barriers to reprogramming within the context of a developing organism.

Along with chromatin structure, co-factors regulate mammalian pioneer-factor binding in culture, including binding by Oct4, Sox2 and FOXA2^12,14,77^. In addition to chromatin accessibility, our data similarly support a role for specific transcription factors in regulating Zld binding in type II neuroblasts. Type II neuroblast-specific Zld-bound loci are not enriched for the canonical Zld motif, suggesting additional factors facilitate Zld binding to these regions. Supporting a functional role for this recruitment, expression of Zld with mutations in the zinc-finger DNA-binding domain, which abrogate sequence-specific binding^24,25^, can still drive supernumerary neuroblasts. We have previously shown that while this mutant protein lacks sequence-specific binding properties, the polypeptide retains an affinity for nucleosomal DNA^57,78^. This nonspecific affinity may be stabilized by additional factors expressed in the neuroblasts. One such factor may be the GA-dinucleotide binding factor CLAMP that has recently been shown to promote Zld binding at promoters and whose binding motif is enriched at neuroblast-specific, Zld-binding sites^79^.

The capacity of Zld to drive the undifferentiated state is limited along the type II neuroblast lineage. While expression of Zld in immature INPs results in supernumerary neuroblasts, Zld expression in mature INPs does not. Similarly, misexpression of the Zld-target gene *dpn* in immature INPs can drive their reversion to neuroblasts, and our data suggest that Zld-mediated reversion is caused, at least in part, by driving expression of *dpn*. By contrast, the endogenous expression of *dpn* in mature INPs does not cause their reversion to neuroblasts because the self-renewal program is decommissioned during INP maturation. This decommissioning is mediated by successive transcriptional repressor activity^41^. We recently demonstrated that Erm and Ham function sequentially to repress expression of genes that promote neuroblast fate. Our data suggest that changes to the chromatin structure mediated by these transcriptional repressors limit the ability of Zld to drive gene expression and therefore reprogram mature INPs (Fig. 7a).

An essential target of Erm and Ham repression is *tll*^41^. In contrast to other stem-cell regulators like Notch and Dpn, Tll is expressed only in type II neuroblasts and not in the transit-amplifying INPs^41,61^. Furthermore, expression of *tll* in mature INPs can robustly drive supernumerary neuroblasts^41,61^. In the embryo, *tll* is a Zld-target gene^28,57^, and our ChIP-seq data identify Zld-binding sites in the type II neuroblasts. While Zld occupies the promoter of *tll* in both type II neuroblasts and the embryo, we identify unique binding sites for Zld in upstream regions in both cell types and demonstrate that these likely denote cell-type-specific enhancers. Our data isolate a neuroblast-specific, Zld-bound enhancer that drives expression specifically in the neuroblasts and supports a model whereby Zld activates expression from this enhancer in the type II neuroblasts. Erm- and Ham-mediate chromatin changes, likely through histone deacetylation, that progressively limit chromatin accessibility during INP maturation. This decrease in accessibility inhibits the ability of ectopically expressed Zld to activate expression from this enhancer, keeping *tll* repressed in the INPs. Gene expression profiling identified *Six4* as a gene that, like *tll*, is expressed only in neuroblasts and not in INPs^41^. Here we identified a neuroblast-specific Zld-bound region that progressively loses accessibility during INP maturation. Thus, Erm and Ham likely silence multiple Zld-bound enhancers to allow for the transition from a self-renewing neuroblast to a transient-amplifying INP. We propose that while the Zld-bound neuroblast-specific enhancer is accessible in neuroblasts, following asymmetric division, changes to the chromatin state mediated by Erm and Ham and downregulation of Zld expression robustly decommissions this enhancer, allowing for differentiation (Fig. 7).

Our data support a model in which Erm- and Ham-mediated changes to the chromatin inhibit binding by the pioneer factor Zld and, in so doing, limit the ability of Zld expression to reprogram cell fate. Our studies in both the early embryo and in type II neuroblasts provide a powerful platform for identifying the barriers to pioneer-factor-mediated reprogramming within the context of development and support a role for both chromatin organization and cell-type-specific co-factors in determining Zld occupancy. Future studies will reveal these specific barriers and will help to identify fundamental processes that may limit reprogramming both in culture and in disease states.

## Methods

### Drosophila strains

Flies were raised on standard fly food at 25 °C (unless otherwise noted). To obtain the *brat*-mutant brains used for ChIP-seq and ATAC-seq, we crossed *brat*^11^*/CyO, ActGFP* to *brat*^*Df(2L)Exel8040*^*/CyO, ActGFP* (BDSC#7847)^80,81^ and screened for GFP negative larvae. Imaging experiments took advantage of a line with endogenous Zld tagged with superfolder GFP^24^. For ChIP-seq experiments using the anti-GFP antibody and GFP-Zld quantification, the sfGFP-tagged Zld alleles were combined with *brat*^11^ and *brat*^*Df(2L)Exel8040*^ and crossed to obtain *brat*-mutant brains. Imaging of Six4::GFP (BDSC#67733) and FlyLight GMR31F04-Gal4 (BDSC#46187) and GMR31D09-Gal4 (BDSC#49676) was done with previously published lines.

For ectopic Zld expression experiments, the open reading frame for Zld-RB was cloned into the pUASt-attB vector using standard PCR, restriction digest and ligation procedures. Similarly, transgenic UAS driven Zld ZnF mutants were cloned using previously generated plasmids^25^ and a gBlock containing ZnF mutations in the DBD of Zld (Integrated DNA Technologies, Coralville, IA). The mutated C2H2 ZnFs convert cysteines to serines. These transgenes were integrated at ZH-86Fb site on chromosome 3 using φC31-mediated integration^82^ (BestGene, Chino Hills, CA). The following drivers were used to drive ectopic expression of Zld in the larval type II neuroblast lineage: *Worniu-Gal4; Asense-Gal80*^83^ combines the *Worniu-Gal4* that expresses in type I and II neuroblasts with the Gal4 inhibitor Gal80 under the *Asense* promoter to obtain a line that drives expression specifically in type II neuroblasts. *Worniu-Gal4; Tub-Gal80ts*^80^ is temperature sensitive thus upon shift from 18 °C to 33 °C, Gal80 repression of Gal4 is relieved and expression is driven. The temperature shift was performed from 24 to 96 h after egg laying, corresponding to larval stages L1-L3. *Erm(II)-Gal4*^47^ drives expression in the Ase- immature INPs through Ase+ immature INPs and mature INPs. *Erm(III)-Gal4*^84^ drives expression in the Ase+ and mature INPs. *Opa-Gal4* (BDSC#46979, *GMR77B05-Gal4*) drives expression in the mature INPs.

To create the *dpn* transgenes we ordered gBlocks (Integrated DNA Technologies, Coralville, IA) with mutated Zld and Su(H) binding sites in the 5 kb *dpn cis*-regulatory and used Gateway LR Clonase (ThermoFisher Scientific, Waltham, MA) to clone the region into the VanGlow vector without the DSCP (Addgene#83342)^50^. The various transgenic reporters were integrated into the VK37 site on chromosome 2 using φC31-mediated integration^82^ (BestGene, Chino Hills, CA). L3 brains from reporter fly lines were dissected, fixed and stained to visualize GFP reporter expression in the type II NBs. Embryos from these lines were collected on molasses plates with yeast paste from flies reared in cages for luciferase assays. The embryos were ground in an eppendorf tube with a pestle in 1x passive lysis buffer and luciferase signal was measured using the Dual Luciferase assay from Promega (Promega, Madison, WI). *tll* transgenes were similarly cloned with Gateway LR Clonase (ThermoFisher Scientific, Waltham, MA) to insert PCR-amplified genomic regions into the VanGlow vector with the DSCP (Addgene#83338). The various transgenic reporters were integrated into the VK31 site on chromosome 3 using φC31-mediated integration^82^ (BestGene, Chino Hills, CA).

*dpn*^172^, *ham*^*Df(2L)Exel7071*^ (BDSC#7843)^41^, and *erm*^*l(2)513843*^ mutant experiments were done using previously published lines. *zld*^294^ ^26^ and Notch-RNAi (BDSC#33611) clones were generated using previously published methods^85^. *Wor-Gal4* was used to drive a GFP reporter to mark neuroblast clones. Briefly, clones were induced by heat shock at 37 °C for 90 min at 24 h after larval hatching. Brains were dissected for clone analysis at 72 h after clone induction.

### Immunofluorescent staining and antibodies

Third instar larval brains were dissected in PBS and fixed in 100 mM Pipes (pH 6.9), 1 mM EGTA, 0.3% Triton X-100 and 1 mM MgSO_4_ containing 4% formaldehyde for 23 min. Fixed brain samples were washed with PBST containing PBS and 0.3% Triton X-100. After removing fix solution, samples were incubated with primary antibodies for 3 h at room temperature. Samples were washed with PBST and incubated with secondary antibodies overnight at 4 °C. The next day samples were washed with PBST and then equilibrated in ProLong Gold antifade mountant (ThermoFisher Scientific, Waltham, MA). The confocal images were acquired on a Leica SP5 scanning confocal microscope (Leica Microsystems Inc, Buffalo Grove, IL). More than 10 brains per genotype were used to obtain data in each experiment. Primary antibodies include Rabbit anti-Ase Antibody (1:400 for IF)^43^, Rat anti-Dpn Antibody (clone 11D1BC7.14; 1:2 for IF)^86^, Rat anti-Wor Antibody (clone CD#72B7AF3; 1:1 for IF)^86^, Chicken anti-GFP Antibody (Aves Labs, Davis, CA, Cat #GFP-1020; 1:2000), and Rhodamin phalloidin (ThermoFisher Scientific, Waltham, MA, Cat #R415; 1:500). Secondary antibodies include Alexa Fluor® 647 AffiniPure Goat Anti-Rat IgG (H + L) (Jackson Immunoresearch, West Grove, PA, Cat#112-605-167; 1:500), Alexa Fluor® 488 AffiniPure Donkey Anti-Chicken IgY (IgG) (H + L) (Jackson Immunoresearch, West Grove, PA, Cat#703-545-155; 1:500), Goat anti-Rabbit IgG (H + L) Highly Cross-Adsorbed Secondary Antibody, Alexa Fluor 488 (ThermoFisher Scientific, Waltham, MA, Cat#A11034; 1:500).

### In situ hybridization chain reaction (HCR) and immunofluorescent staining

mRNA signals in the larval brain were developed by performing in situ HCR v3.0^87^. We modified the protocol of in situ HCR v3.0 to combine immunofluorescent staining of the larval brain. Third instar larval brains were dissected in PBS and fixed in 100 mM Pipes (pH 6.9), 1 mM EGTA, 0.3% Triton X-100 and 1 mM MgSO_4_ containing 4% formaldehyde for 23 minutes. Fixed brain samples were washed with PBST containing PBS and 0.3% Triton X-100. After removing fix solution, samples were pre-hybridized with hybridization buffer (10% formamide, 5×SSC, 0.3% Triton X-100 and 10% dextran sulfate) at 37 °C for 1 h. Pre-hybridized samples were mixed with 5 nM *Sp-1* mRNA HCR probe (Molecular Instruments, Los Angeles, CA) and incubated at 37 °C overnight. After hybridization, samples were washed with washing buffer (10% formamide, 5× SSC, 0.3% Triton X-100) and then incubated with amplification buffer (5× SSC, 0.3% Triton X-100 and 10% dextran sulfate) at 25 °C for 30 min. During washing period, imager hairpins (Molecular Instruments, Los Angeles, CA) were denatured at 95 °C for 2 min. Once samples were equilibrated in amplification buffer, samples were mixed with 3 μM of denatured imager hairpins and incubated at 25 °C for overnight. The next day, samples were washed with PBST and then refixed in 100 mM Pipes (pH 6.9), 1 mM EGTA, 0.3% Triton X-100 and 1 mM MgSO_4_ containing 4% formaldehyde for 15 min to initiate immunofluorescent staining procedures.

### Image quantification

Dpn or Wor were used to identify the type II neuroblast or INP nucleus. The pixel intensities of the reference proteins were measured in nucleus of cells of interest by using Image J software (version 1.53 m) and the pixel intensities of GFP reporter proteins in the identical area were measured. The intensities of GFP signal were corrected based on the difference of intensities of the reference protein in each sample and then the average of GFP intensities for each experiments were calculated. All biological replicates were independently collected and processed.

### Chromatin immunoprecipitation

Five hundred brains (in 45 min time windows) were dissected in Schneider’s medium (Fisher, Hampton, NH, Cat #21720001) from *brat*^*11/Df(2L)Exel8040*^ larvae aged for 5–6 days at 25 °C (L3 stage). The dissected brains were fixed in 1.8% formaldehyde for 20 min, which was stopped by incubation with 0.25 M glycine at room temperature for 4 min and on ice for 10 min. The samples were washed 3 times with wash buffer (1xPBS, 5 mM Tris-HCl pH 7.5, 1 mM EDTA), flash frozen in liquid nitrogen and stored at −80 °C until all the brains had been collected. Brains were thawed on ice and combined for homogenization in SDS lysis buffer (1% SDS, 1 mM PMSF, 50 mM Tris-HCl pH 8.1, 5 mM EDTA) containing protease inhibitors (Pierce mini tablets EDTA-free, VWR, Radnor, PA, Cat #PIA32955) to obtain nuclear extracts. The nuclear extracts were disrupted using a Covaris sonicator (S220 High Performance Ultrasonicator) (18 cycles of 170 Peak Power, 10 Duty Factor, 200 cycles/burst for 60 sec). 7% of the sonicated sample was stored as input and the rest was diluted with 1× volume of dilution buffer (1% Triton X-100, 280 mM NaCl) and incubated overnight with antibodies (8 µl anti-Zld^22^ or 1.4 µl anti-GFP (Abcam, Cambridge, UK, Cat #ab290)). Protein A beads (Dynabeads Protein A, ThermoFisher Scientific, Waltham, MA, Cat #10002D) were added and samples were incubated at 4 °C for 4 h. Beads were recovered and washed. (1× with low salt wash buffer (0.1% SDS, 1% Triton X-100, 2 mM EDTA, 20 mM Tris-HCl pH 8.1 and 150 mM NaCl), 1× high salt wash buffer (0.1% SDS, 1% Triton X-100, 2 mM EDTA, 20 mM Tris-HCl pH 8.1, 500 mM NaCl), 1× LiCl wash buffer (0.25 M LiCL, 1% NP40, 1% deoxycholate, 1 mM EDTA, 10 mM Tris-HCl pH 8.1), and 2× with TE buffer (10 mM Tris-HCl pH 8.0, 1 mM EDTA).) Washed beads were incubated at room temperature for 15 min with elution buffer (0.1 M NaHCO_3_, 1% SDS) to elute the chromatin. The samples and corresponding input were incubated at 55 °C overnight with PK solution (15 µl 1 M Tris pH 7.5, 7 µl 0.5 M EDTA, 4 µl 5 M NaCl, 2 µl 20 mg/ml Proteinase K (Fisher, Hampton, NH, Cat #EO0491)). Samples were incubated with 0.5 µl 20 mg/ml RNase (PureLink RNase A, Thermo Fisher, Waltham, MA, Cat #12091021) at 37 °C for 30 min and moved to 65 °C for 6 h to reverse the crosslinking. Samples were cleaned up by phenol:chloroform extraction, precipitated, and resuspended in 20 µl TE buffer. Sequencing libraries were made using the NEB Next Ultra II DNA library kit (New England BioLabs Inc, Ipswich, MA, Cat #E7645S) and were sequenced on the Illumina Hi-Seq4000 using 50 bp single-end reads at the Northwestern Sequencing Core (NUCore).

### ChIP-seq data analysis

Read quality was checked using FASTQC (version 0.11.9)^88^. Adapters and low-quality bases were removed using Trimmomatic-0.39.^89^. Reads were mapped to the dm6 genome assembly^90^ using Bowtie 2^91^. Unmapped, multiply aligning, mitochondrial, and scaffold reads were removed. Throughout SAMtools was used to filter and convert file formats^92^. MACS version 2^93^ was used with default parameters to identify bound regions of chromatin in samples (IP vs INPUT) for both replicates of Zld antibody ChIP in neuroblasts. The Zld antibody ChIP in the embryo did not have a corresponding INPUT for the single IP, so peaks were called without reference to a control. The GFP antibody ChIP in the neuroblasts was called with the parameters above with the exception of lowering the m-fold value to -m 3 50 due to low IP efficiency. Peak summits were extended by 100 bps on either side. High-confidence Zld-bound regions in the neuroblasts were called as 200 bp peak regions with 50% overlap in both replicates using the BEDtools (version 2.29.0) intersect function^94^. Regions belonging to contigs and unmapped chromosomes were removed. High-confidence regions used for analysis with the GFP antibody ChIP in neuroblasts were called as being bound with 50% overlap in both Zld antibody replicates and the GFP antibody replicate. Comparison of Zld-binding sites between the embryo and neuroblasts were performed by intersecting high-confidence bound regions from the Zld antibody ChIP in the embryo and Zld antibody ChIP in the neuroblasts. Shared regions had 10 bp overlap between the embryo and neuroblasts, and unique regions had less than 10 bp overlap (Supplementary Data 1). Visualization of genomic data was achieved by generation of z score-normalized bigWig files^95^ from merged read coverage of replicates and displayed using Gviz^96^ and the UCSC Genome Browser (http://genome.ucsc.edu^97,98^. z score-normalized bigWigs were created by subtracting the mean read coverage (counts) from merged replicate read counts in 10 bp bins across the entire genome and dividing by the standard deviation.

Heatmaps were generated using deepTools2^99^ with z score-normalized bigWig files. Average signal line plots were generated using seqplots from z score-normalized bigWig files at 10 base-pair resolution^100^. Genomic annotations were performed with the Bioconductor R package ChIPseeker (Bioconductor version 3.9, ChIPseeker version 1.18.0) using default settings and the BDGP dm6 genome through TxDb.Dmelanogaster.UCSC.dm6.ensGene package (BDGP version 1.4.1, TxDb version 3.4.4). TSS regions were redefined as −500 to +150 bps. Peak to nearest gene assignments were done using default settings of the annotatePeak() function.

To test for enrichment of motifs, de novo motif searches were done using the MEME-suite (version 5.1.1)^101^ and Hypergeometric Optimization of Motif Enrichment (HOMER, version 4.11)^102^. These programs identified motifs enriched in the input relative to shuffled or randomized regions of the genome. The de novo motifs were matched to known motifs from the JASPAR (http://jaspar.genereg.net) and DMMPMM (http://autosome.ru/DMMPMM) databases by the programs. Motifs are given a *p*-value or E-value (the *p*-value multiplied by the number of candidate motifs tested) indicating the confidence of the enrichment relative to the control sequences. Additional motif searches were done using the Biostrings package in R (version 2.50.2). The vcountPattern() function was used to tally the number of regions containing at least one occurrence of a motif and the vmatchPattern() was used to locate regions containing a motif.

To assess ChIP-seq and ATAC-seq replicate reproducibility, the number of reads overlapping each peak was quantified using featureCounts (version 1.6.4)^103^. Log_2_(counts) overlapping each peak was plotted and the Pearson correlation calculated for each pair of replicates.

### Assay for transposase-accessible chromatin

The protocol for ATAC-seq on larval brains was adapted from a protocol previously used for ATAC-seq on single embryos^104^. *brat*^*11/Df(2L)Exel8040*^ larvae were aged for 5–6 days at 25 °C prior to dissecting brains in Schneider’s medium (Fisher, Hampton, NH, Cat # 21720001). 5 dissected brains were transferred to the detached cap of a microcentrifuge tube containing 10 µL cold lysis buffer (10 mM Tris, pH 7.5; 10 mM NaCl; 3 mM MgCl_2_; 0.1% NP40). Under a dissecting microscope, brains were homogenized with the blunted tip of a pasteur pipette. The cap was transferred to a microcentrifuge tube containing 40 µL of additional lysis buffer. Tubes were spun down for 10 min at 500 × *g* at 4 °C. Supernatant was removed under a dissecting microscope. This nuclear pellet was used to prepare ATAC-seq libraries using the Nextera DNA Library Preparation Kit (Illumina, San Diego, CA, Cat #FC1211030). The pellet was suspended in 5 µL buffer TD before adding 2.5 µL water and 2.5 µL Tn5 transposase (Tagment DNA Enzyme, Illumina, San Diego, CA, Cat# 15027865). Samples were incubated at 37 °C for 30 min. Tagmented DNA was purified using the Minelute Cleanup Kit (Qiagen, Hilden, DE, Cat #28004). DNA was amplified with 12 cycles of PCR using the NebNext Hi-Fi 2× PCR Master Mix (New England Biolabs Inc, Ipswich, MA, Cat #M0541S). Following PCR, DNA was purified using a 1.2× ratio of Axyprep magnetic beads (Axygen, Corning, NY, Cat #14223151). Library quality and tagmentation were assessed on an Agilent Bioanalyzer before pooling and submitting for 150 bp paired-end sequencing on a Illumina HiSeq 4000 at NovoGene.

Chromatin accessibility during the transition from type II neuroblast to INP was characterized using a temperature-sensitive system for synchronous type II neuroblast differentiation (Rives-Quinto et al. 2020). Brains were collected at 0, 6, 12, 18, or 24 h following temperature shift, capturing intermediate stages during the transition from type II neuroblasts to INP. Following temperature shift, brains were collected and ATAC-seq libraries prepared as described above.

### ATAC-seq analysis

Adapter sequences were trimmed from raw sequence reads using NGmerge (version 0.3)^105^. Reads were aligned to the *D. melanogaster* genome (version dm6) using Bowtie2 using the following parameters: --very-sensitive, --no-mixed, --no-discordant, -X 5000, -k 2^91^. Aligned reads were filtered to include only reads with a mapping quality score >30. Reads aligning to scaffolds or the mitochondrial genome were discarded. To identify fragments that likely originated from nucleosome-free regions, fragments were filtered to include only those <100 bp, as previously described^106^. All downstream analysis and visualization was performed using these accessible fragments. To call peaks on accessible fragments, accessible fragments from both replicates were merged and MACS2 was used with the following parameters: -f BAMPE, --keep-dup all, -g 1.2e8, --call-summits. Deeptools was used to calculate genome coverage and generate bigWig files used for genome browser tracks and metaplots.

To compare Zld binding to chromatin accessibility in the embryo and neuroblasts, neuroblast-specific, embryo-specific and shared Zld peaks were merged to create a set of all Zld peaks detected in either cell type. The number of ChIP-seq or ATAC-seq reads overlapping each peak was quantified using featureCounts for the following datasets: neuroblast Zld ChIP, nuclear cycle 14 embryo Zld ChIP^22^, neuroblast ATAC and stage 5 embryo ATAC^59^. DESeq2 was used to compare ChIP-seq or ATAC-seq profiles between the embryo and neuroblast and identify differentially bound or differentially accessible regions between the two cell types^107^. Log_2_ fold-change values calculated by DESeq were used to correlate differences in binding with differences in accessibility between the two cell types.

To identify regions with dynamic chromatin accessibility during the transition from type II neuroblast to INP, DEseq was used to perform differential accessibility analysis across data from the 5 ATAC-seq time points. The likelihood ratio test was used to identify 11,127 regions with differential accessibility across any of the time points. K-means clustering was performed to separate sites into groups with different patterns of chromatin accessibility over the time course. We initially tested values of k from 2 to 15 and found that 6 clusters were sufficient to capture the following patterns of change in the dataset: early increase, late increase, transient increase, early loss, late loss, transient loss. Clusters obtained using values of k larger than 6 did not identify additional patterns of change. K-means clustering was performed in R with the following parameters: nstart = 25, max.inter = 1000. For analysis of Zld motifs at differentially accessible sites, regions were considered to contain a Zld motif based on presence of the CAGGTA motif within 200 bp of the peak summit (Supplementary Data 3).

### Reporting summary

Further information on research design is available in the Nature Research Reporting Summary linked to this article.

## Supplementary information

Supplementary Information

Peer Review File

Description of Additional Supplementary Files

Supplementary Data 1

Supplementary Data 2

Supplementary Data 3

Reporting Summary

## Data Availability

The data that support this study are available from the corresponding authors upon reasonable request. Sequencing data generated for this manuscript have been deposited in GEO under accession code GSE150931. Unnormalized bigwig files and peak bed files for all datasets generated are also available under accession code GSE150931. Zld-bound peak regions in the early embryo and type II neuroblasts are located in Supplementary Data 1. Genes associated with Zld binding in the embryo and type II neuroblasts identified by ChIP-seq are located in Supplementary Data 2. Regions of dynamic accessibility during type II neuroblast differentiation identified by ATAC-seq are located in Supplementary Data 3. Source data can be found in the Source Data file. Previously published sequencing data for Zld ChIP-seq in the early embryo can be found in GEO under accession code GSE30757. Sequencing data for ATAC-seq done in the early embryo can be found in GEO under accession code GSE137075. Source data are provided with this paper.

## Source data

Source Data
