# Supplementary material for: Cell-type-specific chromatin occupancy by the pioneer factor Zelda drives key developmental transitions in Drosophila
Source: Nat Commun. 2021 Dec 9;12:7153. doi: 10.1038/s41467-021-27506-y (PMC8660810; doi:10.1038/s41467-021-27506-y)
Supplement: Supplementary file 1 — Supplementary Information [file 41467_2021_27506_MOESM1_ESM.pdf]

Supplementary Information

Cell-type-specific chromatin occupancy by the pioneer factor Zelda drives key developmental transitions in Drosophila

Elizabeth D. Larson, Hideyuki Komori, Tyler J. Gibson, Cyrina M. Ostgaard, Danielle C. Hamm, Jack M. Schnell, Cheng-Yu Lee and Melissa M. Harrison

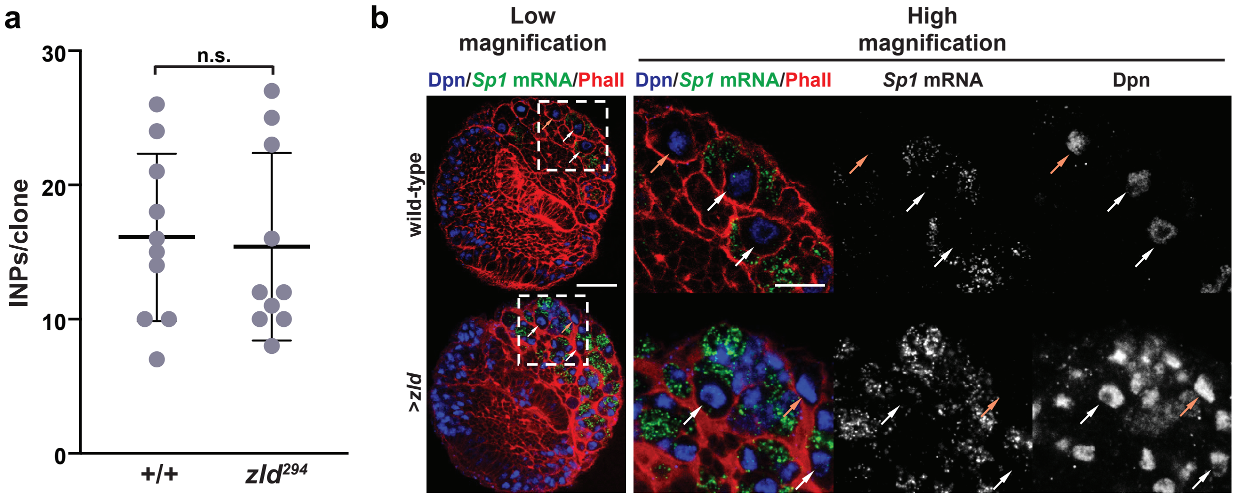

Supplementary Fig. 1 ***zld*-null mutant neuroblast clones are indistinguishable from wild-type, and neuroblasts formed upon overexpression of *zld* are type II neuroblasts.** **a** Number of INPs in either wild-type or *zld*^{294} type II neuroblast clones as marked by Dpn and Ase expression (Dpn+Ase+) . +/+ = 16.1 ± 6.2 INPs and *zld*^{294} = 15.4 ± 7 INPs; n = 10 clones per genotype. Mean number of INPs per clone is shown, and error bars show the standard deviation for a sample. Comparison done using an unpaired two-tailed Student's t-test; n.s. p-value = 0.8160. Source data are provided as a Source Data file. **b** *In situ* hybridization for *Sp1* mRNA in wild-type brains (n = 10 brains) and brains upon overexpression of *zld* with the *Wor-Gal4, TubGal80ts* driver (n = 12 brains) at low (left) and boxed region at high (right) magnification. Staining for Dpn and PhalI markers are also included. Neuroblasts produced upon overexpression of *zld* are type II neurobasts (white arrows). Type I neuroblasts are indicated by the orange arrows. Scale bar, 20µm (left), 10µm (right).

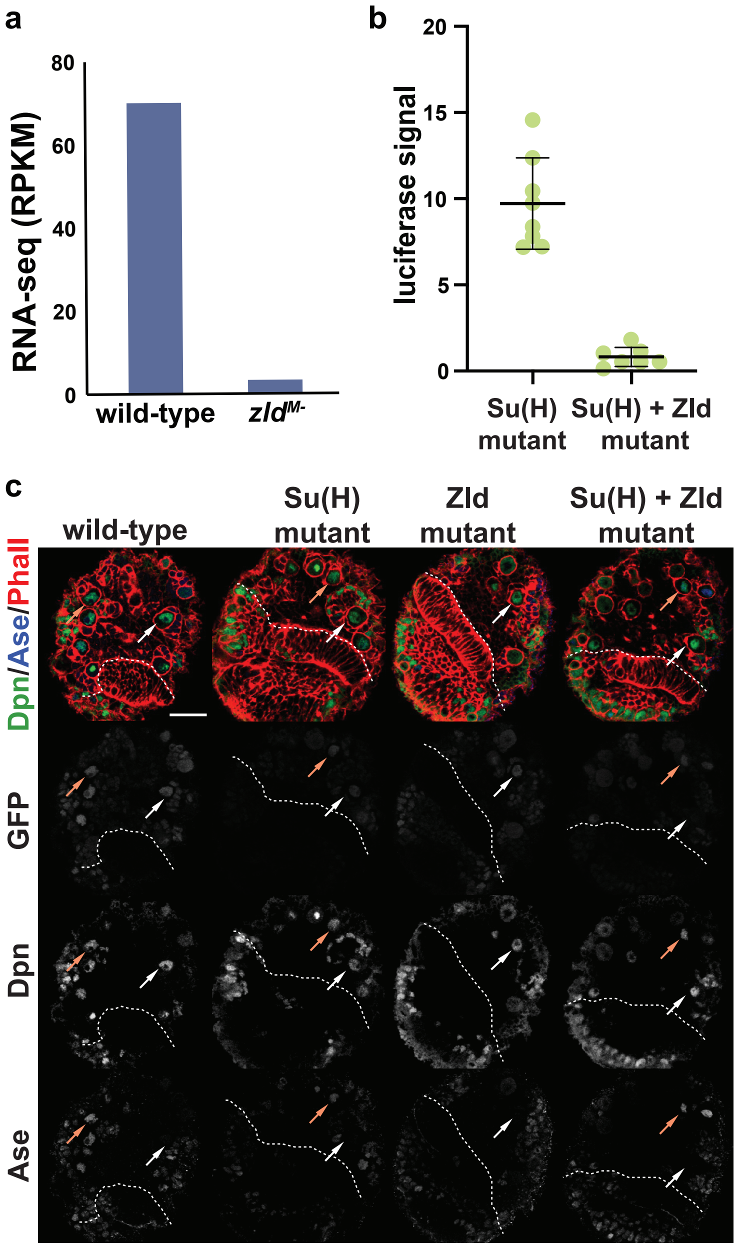

Supplementary Fig. 2 ***dpn* is a Zld-target gene in the early embryo and neuroblasts. a** *dpn* mRNA levels decrease in stage 5 embryos lacking maternally deposited *zld* (*zld*$^{M-}$) compared to wild-type[23]. **b** Luciferase expression of the *dpn* GFP:luciferase reporters in 2-3 hr AEL (after egg laying) embryos require Zld-binding sites for expression (Su(H) mut, n = 8; Su(H) + Zld mut, n = 7). Mean normalized luciferase signal is shown, and error bars show the standard deviation for a sample. Source data are provided as a Source Data file. **c** Whole brain images of GFP expression (second row) in type II neuroblasts of animals expressing transgenes containing the wild-type binding sites (n = 10), mutated Su(H)-binding sites (n = 7), mutated Zld-binding sites (n = 9), or mutated Su(H)- and Zld-binding sites (n = 8). Staining for markers of the neuroblasts are also shown. White arrows point to type II neuroblasts,- and orange arrows points to type I neuroblasts. White dashed line highlights the division between the optic lobe and the brain. Scale bar, 20μm.

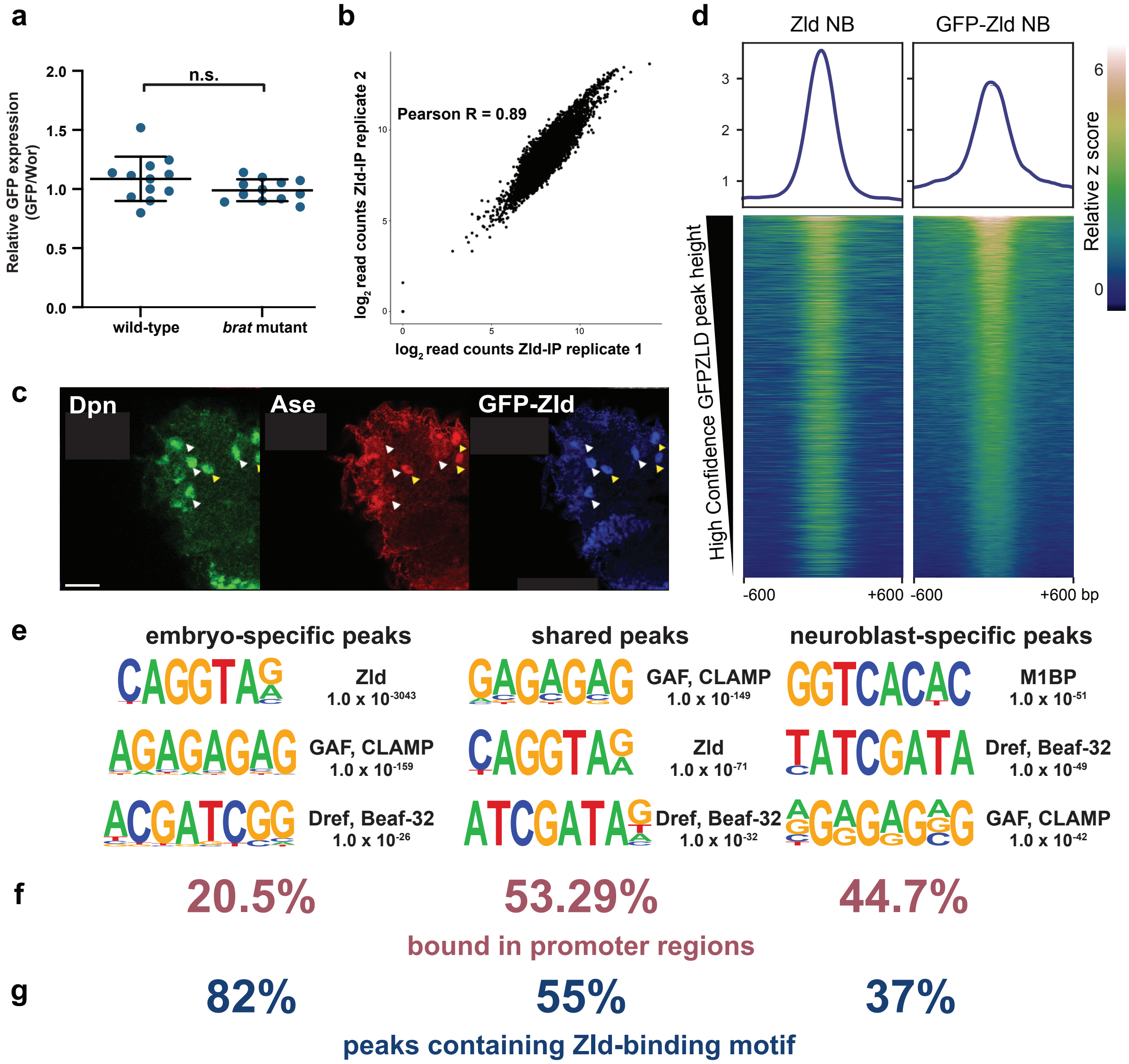

Supplementary Fig. 3 **ChIP-seq for GFP-Zld confirms neuroblast-specific Zld-bound regions.** **a** Relative quantification of endogenous GFP-Zld levels in type II neuroblasts of wild-type and *brat*$^{11/Df(2L)Excel8040}$ brains as compared to Wor expression. n = 12 neuroblasts per genotype. Mean relative GFP expression is shown, and error bars show the standard deviation for a sample. Comparison done using an unpaired two-tailed Student's t-test; n.s. p-value = 0.1237. Source data are provided as a Source Data file. **b** Pearson correlation plot of read coverage for the two Zld-antibody replicates for ChIP-seq from *brat*$^{11/Df(2L)Excel8040}$ brains shows high correlation between replicates. **c** Third instar larval brain stained for Ase, Dpn and GFP-Zld. GFP-Zld is expressed in type I neuroblasts (yellow arrowhead) and type II neuroblasts (white arrowhead). Scale bar, 20µm. **d** Heat maps centered on the ChIP peak with 600bp flanking sequence for peaks bound by the anti-Zld antibody (Zld NB) and the anti-GFP antibody (GFP-Zld NB). Colors indicate relative ChIP z score. Average z score is shown above each heat map. The highest bound regions identified by the Zld antibody are also the highest bound regions identified by the GFP antibody. **e** Motif enrichment for the high-confidence embryo-specific peaks, shared peaks and neuroblast-specific peaks identified by both Zld and GFP antibodies as determined by HOMER. The same motifs are enriched as when only regions bound by the Zld antibody are analyzed. **f** Fraction of high-confidence Zld-bound regions (identified using anti-Zld and anti-GFP antibody) bound in promoters (-500bps to +150bps). There is an enrichment for Zld binding to promoters in the regions specific to the neuroblasts and shared regions between the embryo and neuroblasts. **g** Percent of peaks in each class that contain a variant of the Zld-binding motif (NAGGYAN). The Zld-binding motif is more enriched in regions specific to the embryo and shared regions.

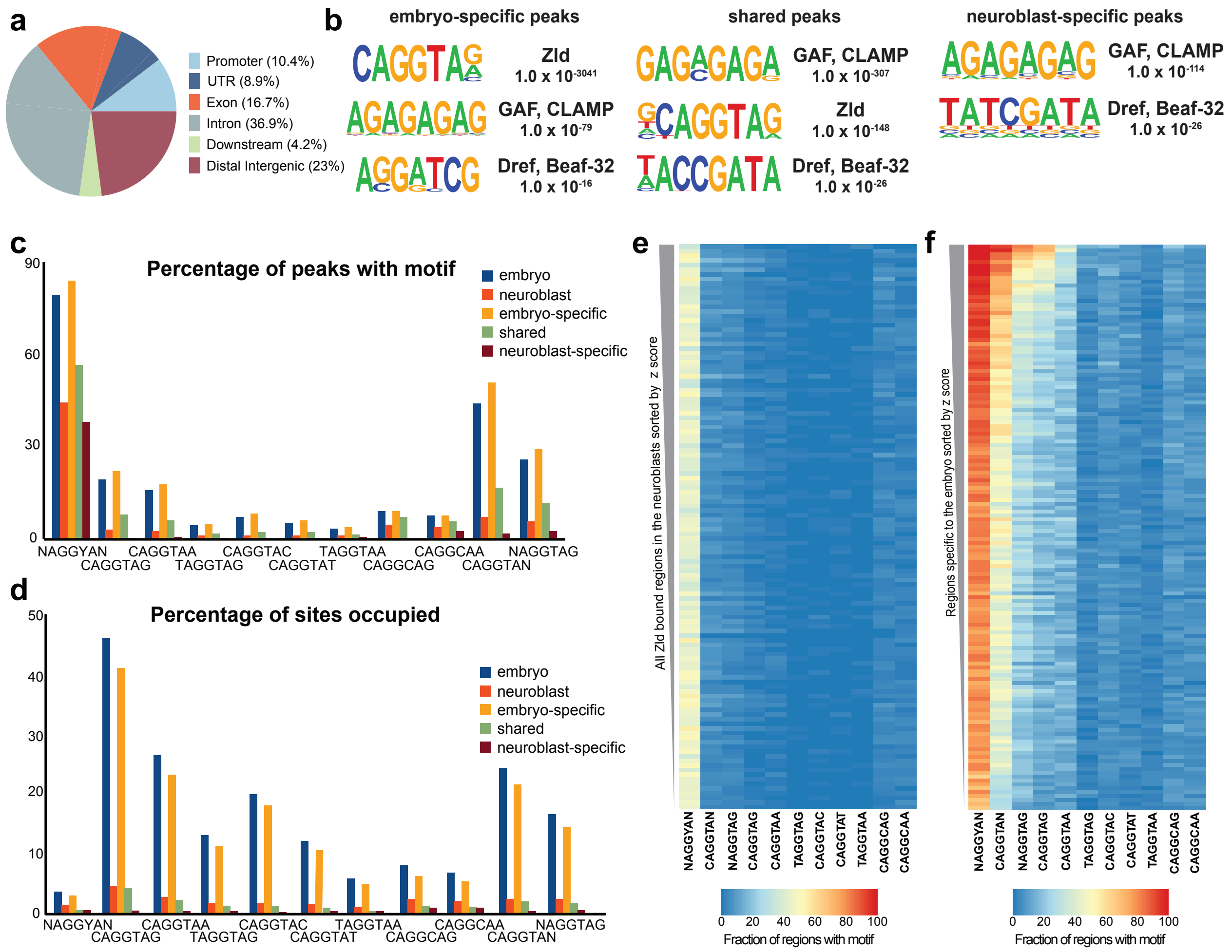

Supplementary Fig. 4 **The canonical Zld-binding motif is not enriched in regions specifically bound by Zld in the neuroblasts. a** Genomic distribution of random 200bp regions of the *Drosophila* genome (shuffled unique neuroblast Zld-bound regions). Promoter regions defined as -500bps to +150bps from the TSS. **b** Motif enrichment for the high-confidence embryo-specific peaks, shared peaks and neuroblast-specific peaks using the anti-Zld antibody as determined by HOMER. The canonical Zld-binding motif is not enriched in regions specific to Zld binding in the neuroblasts. **c** Percent of peaks containing the Zld-binding motif variant indicated for each class of Zld-bound peaks. **d** Percent of motif variants in the genome occupied in each class of Zld-bound regions. **e** Enrichment of Zld-binding motif variants in all Zld-bound regions identified in type II neuroblasts. Peaks are sorted from highest (top) to lowest (bottom) z score and binned into groups of 100. Color of the cell represents the percent of peaks in each bin containing the indicated motif within the 200bp peak region. There is very low enrichment of all variants. The most enriched variant is a degenerate motif encompassing all variations on the Zld-binding motif (NAGGYAN). **f** Enrichment of Zld-binding motif variants in all Zld-bound regions specific to the early embryo. Peaks are sorted from highest (top) to lowest (bottom) z score and binned into groups of 100. Color of the cell represents the percent of peaks in each bin containing the indicated motif within the 200bp peak region. Both the degenerate motif (NAGGYAN) and the specific variant (CAGGTAN) are highly enriched within these embryo-specific peaks and this enrichment correlates with peak z score.

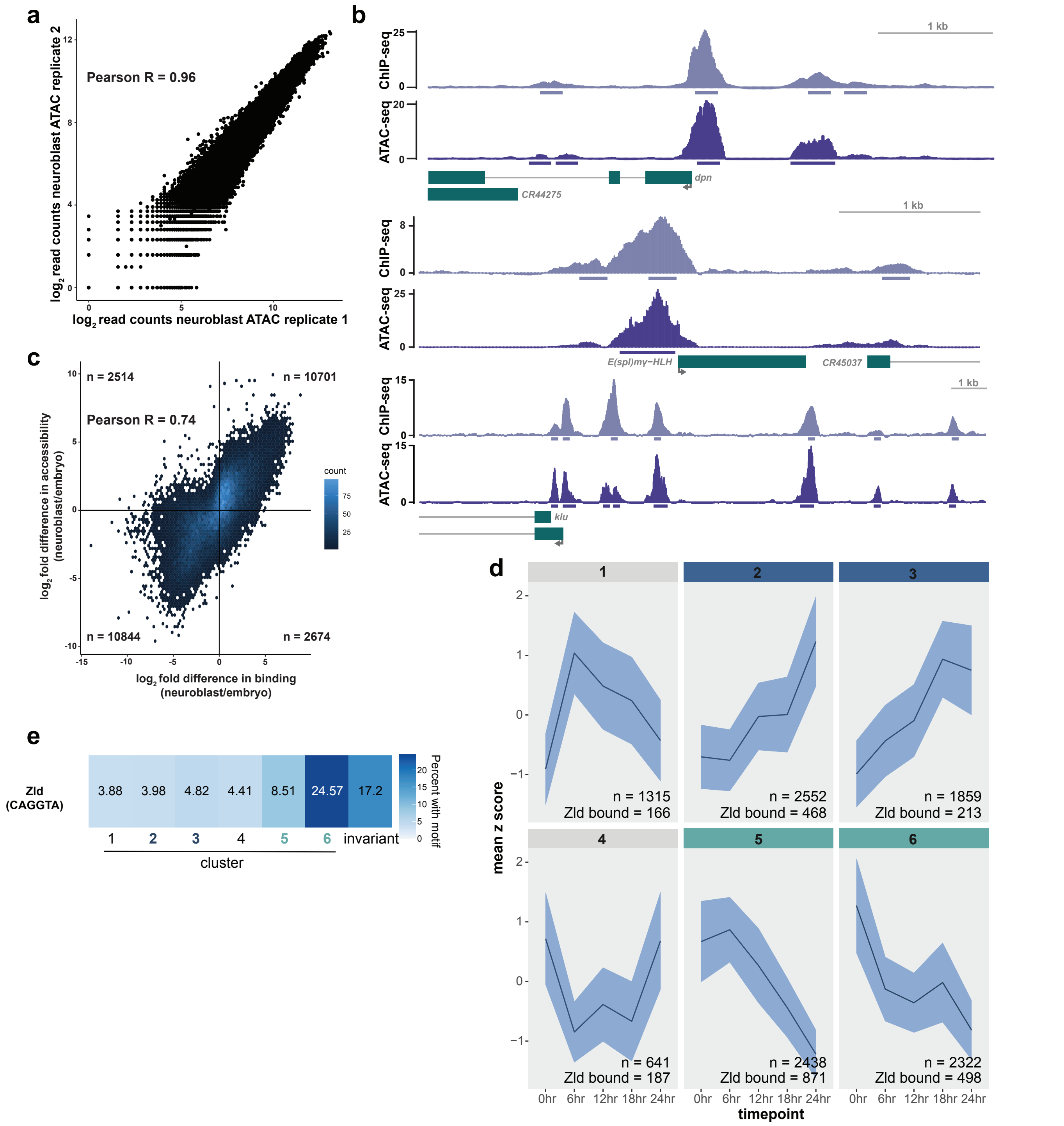

Supplementary Fig. 5 **Zld binding correlates with chromatin accessibility in the embryo and type II neuroblasts. a** Pearson correlation plot of read coverage for ATAC-seq replicates on *$brat^{11/Df(2L)Excel8040}$* brains on a log_2 scale shows high correlation between replicates. **b** Genome browser tracks of Zld binding (ChIP-seq) and chromatin accessibility (ATAC-seq) from type II neuroblasts at the *dpn, E(spl)mγ-HLH* and *klu* loci. Peak regions are shown below the tracks. **c** Differences between Zld binding (ChIP-seq) in the embryo and type II neuroblasts correlate with differences in chromatin accessibility. log_2 fold difference in Zld binding on the x-axis compared to log_2 fold difference in chromatin accessibility on the y-axis. Color represents relative count. **d** k-means clustering of regions that change in accessbility based on ATAC-seq mean z score signal during type II neuroblast differentiation. The shaded areas represent the standard deviation from the mean. Classes 1 and 4 have transient changes in accessibility, classes 2 and 3 increase in accessibility during differentiation and classes 5 and 6 decrease in accessibility. Zld-bound sites in the neuroblasts are enriched in decreasing clusters 4, 5 and 6. **e** The percent of regions in each cluster that contains the Zld motif is indicated by a shade of blue. The canonical Zld-binding motif is enriched in cluster 6.

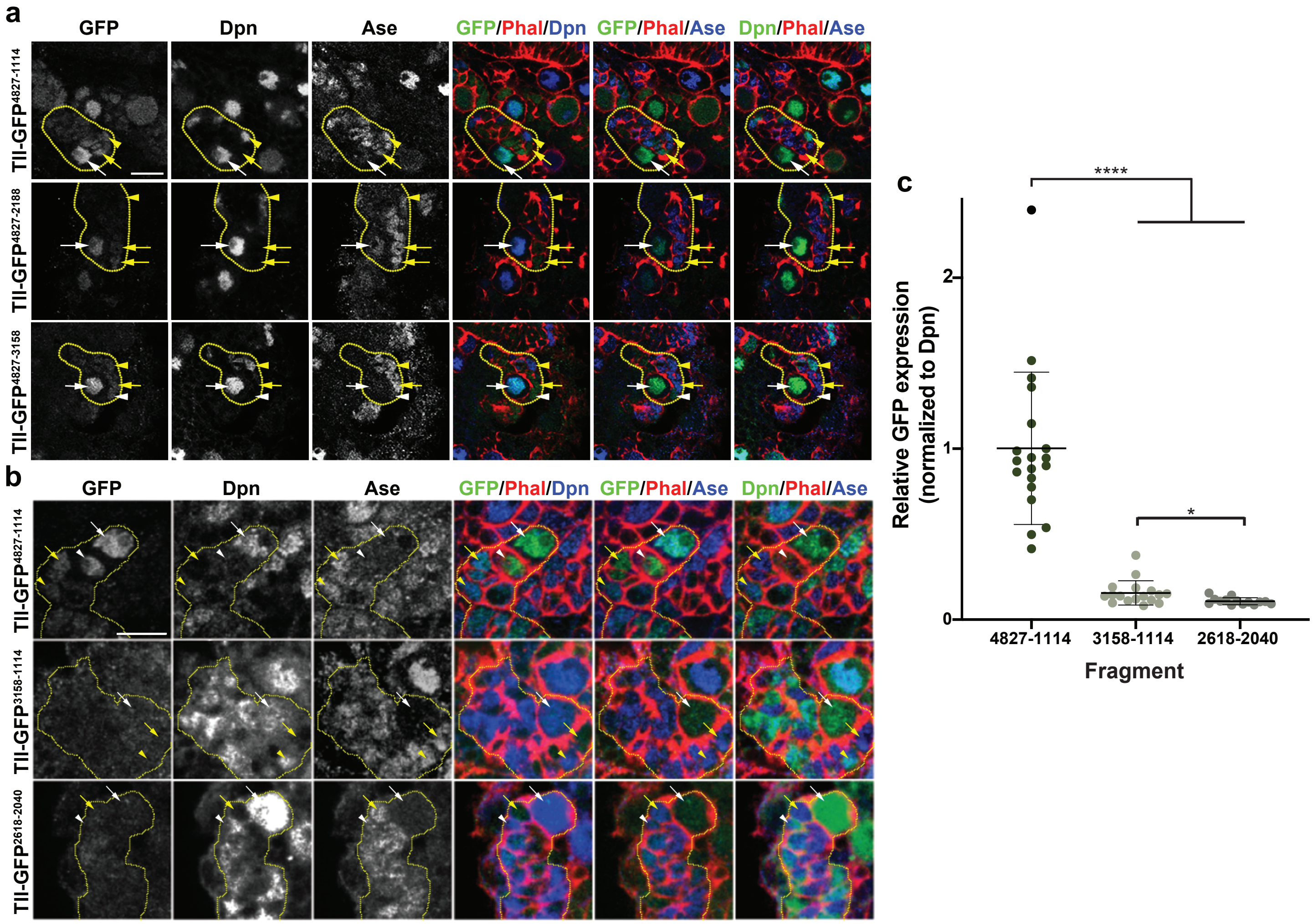

Supplementary Fig. 6 **A neuroblast-specific Zld-bound *tll* enhancer drives reporter expression in neuroblasts. a,b** Representative images of GFP expression in type II neuroblasts of animals expressing transgenes containing truncations of the upstream regulatory region of *tll* with the DSCP driving a GFP reporter. Tll-GFP$^{4827\text{-}1114}$ n = 28 neuroblasts, Tll-GFP$^{4827\text{-}2188}$ n = 45 neuroblasts, Tll-GFP$^{4827\text{-}3158}$ n = 48 neuroblasts. Staining for markers of neuroblasts are also shown. Type II neuroblast lineages are outlined with a dashed yellow line. Yellow arrowheads represent INPs, white arrowheads represent Ase- immature INP, yellow arrows represent Ase+ immature INP and white arrows represent type II neuroblasts. Scale bar, 10μm. **c** Quantification of relative GFP expression in type II neuroblasts for the fragments corresponding to the region indicated below and shown in **b** (normalized to Dpn signal). For fragment 4827-1114 n = 19 neuroblasts, fragment 3158-1114 n = 17 neuroblasts, fragment 2618-2040 n = 14 neuroblasts. Mean relative GFP expression is shown, and error bars show the standard deviation for a sample. Significance with a one-way ANOVA with post hoc Dunnett's multiple comparison test; **** p<0.0001, * p=0.044. Source data are provided as a Source Data file.

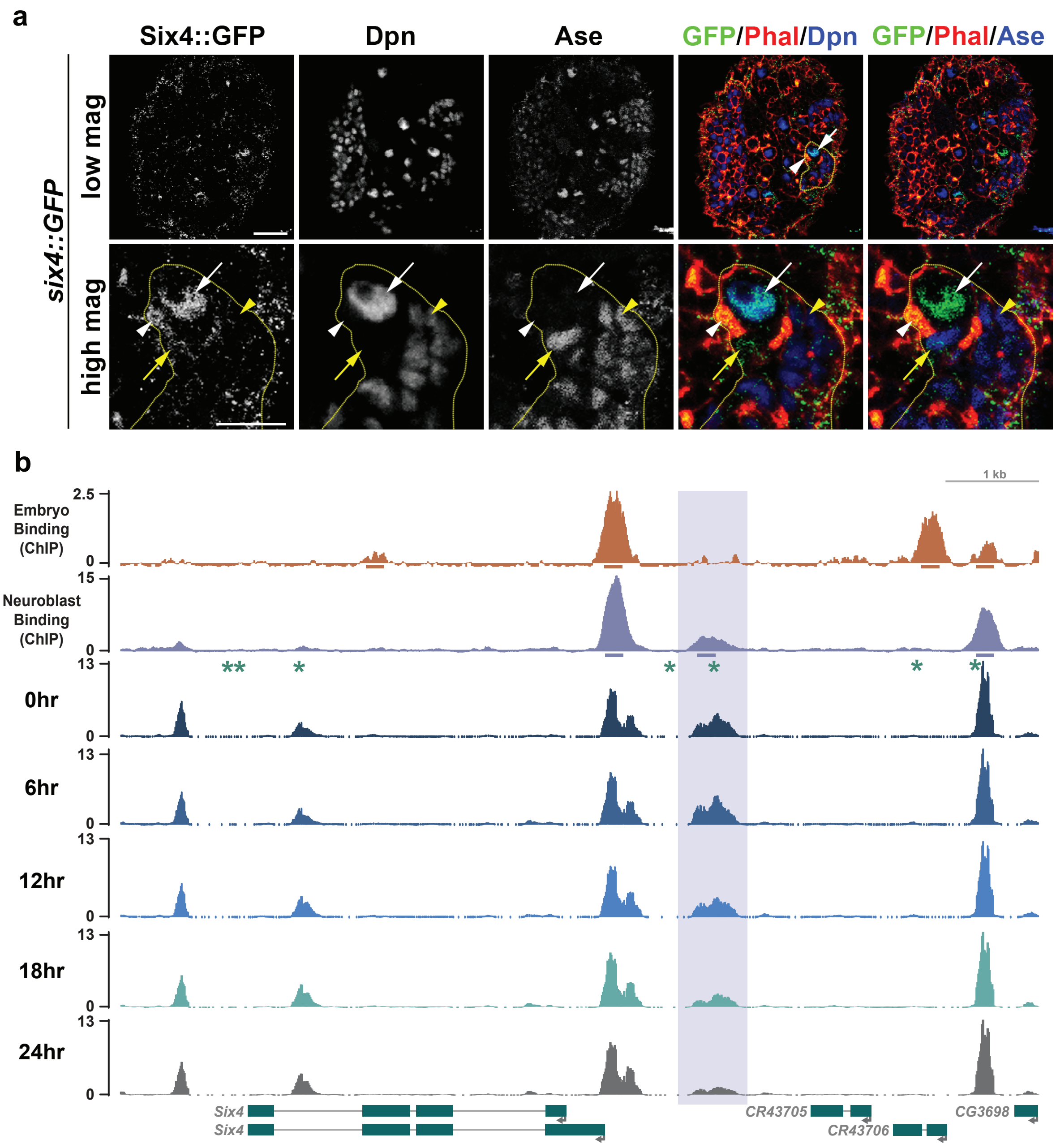

Supplementary Fig. 7 **Six4 expression is limited to the neuroblasts, and a neuroblast-specific Zld bound *Six4* enhancer loses accesibility throughout differentiation. a** Representative images of GFP expression in type II neuroblasts of animals expressing a *Six4::GFP* transgene (n = 15 brains) at low (top) and high (bottom) magnification. Staining for markers of neuroblasts are also shown. Type II neuroblast lineages are outlined with a dashed yellow line. Yellow arrowheads represent INPs, white arrowheads represent Ase- immature INP, yellow arrows represent Ase+ immature INP and white arrows represent type II neuroblasts. Scale bar, 20µm (top), 10µm (bottom). **b** Genome browser tracks of Zld ChIP-seq from the early embryo and type II neuroblasts and ATAC-seq of the *Six4* locus from *brat* mutant brains at the indicated time points following a temperature shift that initiates synchronous differentiation. 200 bp regions surrounding the ChIP peak summits are shown below the top two tracks, and Erm binding motifs (AAAWGVVCMNH) are indicated by the green astrick.
