# Supplementary material for: Cell-type-specific chromatin occupancy by the pioneer factor Zelda drives key developmental transitions in Drosophila
Source: Nat Commun. 2021 Dec 9;12:7153. doi: 10.1038/s41467-021-27506-y (PMC8660810; doi:10.1038/s41467-021-27506-y)
Supplement: Supplementary file 3 — Description of Additional Supplementary Files [file 41467_2021_27506_MOESM3_ESM.pdf]

Description of additional supplementary data files

Supplementary Data 1. Zld bound peak regions in the early embryo and type II neuroblasts. All peaks called for Zld ChIP-seq in the type II neuroblasts and the early embryo, shared peaks and unique peaks to either the neuroblasts or embryo (as indicated in each tab). The chromosome, start and stop position, genomic annotation and associated gene information are included for each peak.

Supplementary Data 2. Genes associated with Zld binding in the embryo and type II neuroblasts identified by ChIP-seq. Gene ID and gene name for all genes bound by Zld in the type II neuroblasts, all genes bound by Zld the early embryo, shared genes, genes unique to Zld bound regions in the neuroblasts and embryo and those genes bound by both Zld and Notch in the neuroblasts (indicated in each tab).

Supplementary Data 3. Regions of dynamic accessibility during type II neuroblast differentiation identified by ATAC-seq. All regions that change in accessibility by ATAC-seq during the induced differentiation time-course. The chromosome, start and stop position, if the region is bound by Zld in the neuroblasts and k-means cluster are provided for each region.
