# Supplementary material for: Cell-type-specific chromatin occupancy by the pioneer factor Zelda drives key developmental transitions in Drosophila
Source: Nat Commun. 2021 Dec 9;12:7153. doi: 10.1038/s41467-021-27506-y (PMC8660810; doi:10.1038/s41467-021-27506-y)
Supplement: Supplementary file 7 — Reporting Summary [file 41467_2021_27506_MOESM7_ESM.pdf]

nature research

Corresponding author(s): Melissa Harrison; Cheng-Yu Lee

Last updated by author(s): Oct 1, 2021

Reporting Summary

Nature Research wishes to improve the reproducibility of the work that we publish. This form provides structure for consistency and transparency in reporting. For further information on Nature Research policies, see our Editorial Policies and the Editorial Policy Checklist.

Statistics

For all statistical analyses, confirm that the following items are present in the figure legend, table legend, main text, or Methods section.

n/a	Confirmed	
☐	☒	The exact sample size (*n*) for each experimental group/condition, given as a discrete number and unit of measurement
☐	☒	A statement on whether measurements were taken from distinct samples or whether the same sample was measured repeatedly
☐	☒	The statistical test(s) used AND whether they are one- or two-sided<br>*Only common tests should be described solely by name; describe more complex techniques in the Methods section.*
☒	☐	A description of all covariates tested
☐	☒	A description of any assumptions or corrections, such as tests of normality and adjustment for multiple comparisons
☐	☒	A full description of the statistical parameters including central tendency (e.g. means) or other basic estimates (e.g. regression coefficient) AND variation (e.g. standard deviation) or associated estimates of uncertainty (e.g. confidence intervals)
☐	☒	For null hypothesis testing, the test statistic (e.g. *F*, *t*, *r*) with confidence intervals, effect sizes, degrees of freedom and *P* value noted<br>*Give P values as exact values whenever suitable.*
☒	☐	For Bayesian analysis, information on the choice of priors and Markov chain Monte Carlo settings
☒	☐	For hierarchical and complex designs, identification of the appropriate level for tests and full reporting of outcomes
☐	☒	Estimates of effect sizes (e.g. Cohen's *d*, Pearson's *r*), indicating how they were calculated

Our web collection on statistics for biologists contains articles on many of the points above.

Software and code

Policy information about availability of computer code

Data collection

Trimmomatic-0.39
Bowtie 2 v2.3.5
MACS v2
SAMtools v1.11
BEDtools v 2.29.0
deepTools2
Gviz R package v 1.28.3
SeqPlots R package v 1.22.2
ChIPseeker R package v 1.18.0
MEME-suite v 5.1.1
HOMER v 4.11
Biostrings R package v 2.50.2
R v 3.6.2
featureCounts R package v 1.6.4
NGmerge v 0.3
DESeq2 R package v 1.24.0
FASTQC v 0.11.9
Image J v 1.53m

Data analysis

ChIP-seq analysis:
Read quality was checked using FASTQC (version 0.11.9). Adapters and low-quality bases were removed using Trimmomatic-0.39. Reads were

mapped to the dm6 genome assembly using Bowtie 2. Throughout SAMtools was used to filter and convert file formats. MACS version 2 was used with default parameters described below to call peaks. Visualization of genomic data was achieved by generation of z score-normalized bigWig files from merged read coverage of replicates and displayed using Gviz and the UCSC Genome Browser. Heatmaps were generated using deepTools2 with z score-normalized bigWig files. Average signal line plots were generated using SeqPlots from z score-normalized bigWig files at 10 base-pair resolution. Genomic annotations were performed with the Bioconductor R package ChIPseeker (Bioconductor version 3.9, ChIPseeker version 1.18.0) using default settings and the BDGP dm6 genome through TxDb.Dmelanogaster.UCSC.dm6.ensGene package (BDGP version 1.4.1, TxDb version 3.4.4). TSS regions were redefined as -500bps to +150bps. Peak to nearest gene assignments were done using default settings of the annotatePeak() function. To test for enrichment of motifs, de novo motif searches were done using the MEME-suite (version 5.1.1) and Hypergeometric Optimization of Motif Enrichment (HOMER, version 4.11). Additional motif searches were done using the Biostrings package in R (version 2.50.2). The vcountPattern() function was used to tally the number of regions containing at least one occurrence of a motif and the vmatchPattern() was used to locate regions containing a motif.

ATAC-seq analysis
Adapter sequences were trimmed from raw sequence reads using NGmerge (version 0.3). Reads were aligned to the D. melanogaster genome (version dm6) using Bowtie2 using the following parameters: --very-sensitive, --no-mixed, --no-discordant, -X 5000, -k 2. Aligned reads were filtered to include only reads with a mapping quality score > 30. Reads aligning to scaffolds or the mitochondrial genome were discarded. To identify fragments that likely originated from nucleosome-free regions, fragments were filtered to include only those < 100 bp, as previously described. All downstream analysis and visualization was performed using these accessible fragments. To call peaks on accessible fragments, accessible fragments from both replicates were merged and MACS2 was used with the following parameters: -f BAMPE, --keep-dup all, -g 1.2e8, --call-summits. Deeptools was used to calculate genome coverage and generate bigWig files used for genome browser tracks and metaplots. DESeq2 was used to compare ChIP-seq or ATAC-seq profiles between the embryo and neuroblast and identify differentially bound or differentially accessible regions between the two cell types. Log2 fold-change values calculated by DESeq were used to correlate differences in binding with differences in accessibility between the two cell types.

To identify regions with dynamic chromatin accessibility during the transition from type II neuroblast to INP, DEseq was used to perform differential accessibility analysis across data from the 5 ATAC-seq time points. The likelihood ratio test was used to identify regions with differential accessibility across any of the time points. K-means clustering was performed to separate sites into groups with different patterns of chromatin accessibility over the time course. We initially tested values of k from 2 to 15 and found that 6 clusters were sufficient to capture the following patterns of change in the dataset. K-means clustering was performed in R with the following parameters: nstart=25, max.inter=1000. For analysis of Zld motifs at differentially accessible sites, regions were considered to contain a Zld motif based on presence of the CAGGTA motif within 200 bp of the peak summit.

Image quantification
Dpn or Wor were used to identify the type II neuroblast or INP nucleus. The pixel intensities of the reference proteins were measured in nucleus of cells of interest by using Image J software (version 1.53m) and the pixel intensities of GFP reporter proteins in the identical area were measured. The intensities of GFP signal were corrected based on the difference of intensities of the reference protein in each samples and then the average of GFP intensities for each experiments were calculated. All biological replicates were independently collected and processed.

For manuscripts utilizing custom algorithms or software that are central to the research but not yet described in published literature, software must be made available to editors and reviewers. We strongly encourage code deposition in a community repository (e.g. GitHub). See the Nature Research guidelines for submitting code & software for further information.

Data

Policy information about availability of data

All manuscripts must include a data availability statement. This statement should provide the following information, where applicable:

- Accession codes, unique identifiers, or web links for publicly available datasets
- A list of figures that have associated raw data
- A description of any restrictions on data availability

Sequencing data generated for this manuscript has been deposited in GEO under accession code GSE150931 (https://www.ncbi.nlm.nih.gov/geo/query/acc.cgi?acc=GSE150931). Unnormalized bigwig files and peak bed files for all datasets generated are also available under accession code GSE150931. Zld bound peak regions in the early embryo and type II neuroblasts are located in Supplementary Data 1. Genes associated with Zld binding in the embryo and type II neuroblasts identified by ChIP-seq are located in Supplementary Data 2. Regions of dynamic accessibility during type II neuroblast differentiation identified by ATAC-seq are located in Supplementary Data 3. Source data can be found in the Source Data file. Sequencing data for Zld ChIP-seq in the early embryo can be found in GEO under accession code GSE30757 (https://www.ncbi.nlm.nih.gov/geo/query/acc.cgi?acc=GSE30757). Sequencing data for ATAC-seq done in the early embryo can be found in GEO under accession code GSE137075 (https://www.ncbi.nlm.nih.gov/geo/query/acc.cgi?acc=GSE137075).

Field-specific reporting

Please select the one below that is the best fit for your research. If you are not sure, read the appropriate sections before making your selection.

☒ Life sciences ☐ Behavioural & social sciences ☐ Ecological, evolutionary & environmental sciences

For a reference copy of the document with all sections, see nature.com/documents/nr-reporting-summary-flat.pdf

Life sciences study design

All studies must disclose on these points even when the disclosure is negative.

Sample size	No statistical approach was used to predetermine sample size. Samples sizes were determined following best practices in the field and our previous experience (Weng M., et al. Dev. Cell, 2010, Development 2012, Komori H., et al Genes & Dev. 2018 Rives-Quinto N., et al. eLife 2020). To quantify the number of specific cell types in the larval brain, eight brain lobes are sufficient to show reproducibility and significance. To quantify the signal intensity of reporter protein expression, seven cells are sufficient to show reproducibility and significance.
Data exclusions	No data were excluded from the analyses.
Replication	Experiments were performed with multiple independent measures. Immunostaining experiments were performed at least three times to confirm reproducibility. Genomic experiments were performed with a minimum of two replicates. Similar results were required in all biological replicates to support or reject the hypothesis. The number of replicates are indicated throughout. All attempts at replication confirmed the reported findings.
Randomization	The only randomization used in this study was the random selection of age and genotype matched larvae in each experimental group.
Blinding	Blinding was performed in counting the number of cells and in quantifying reporter protein expression. Blinding was not used for ChIP-seq and ATAC-seq because as it would not be possible to collect the samples or perform the data analysis.

Reporting for specific materials, systems and methods

We require information from authors about some types of materials, experimental systems and methods used in many studies. Here, indicate whether each material, system or method listed is relevant to your study. If you are not sure if a list item applies to your research, read the appropriate section before selecting a response.

Materials & experimental systems

n/a	Involved in the study	
☐	☒	Antibodies
☒	☐	Eukaryotic cell lines
☒	☐	Palaeontology and archaeology
☐	☒	Animals and other organisms
☒	☐	Human research participants
☒	☐	Clinical data
☒	☐	Dual use research of concern

Methods

n/a	Involved in the study	
☐	☒	ChIP-seq
☒	☐	Flow cytometry
☒	☐	MRI-based neuroimaging

Antibodies

Antibodies used	Rabbit Anti-Ase: Purified in Cheng-Yu Lee Lab<br>Rat Anti-Dpn: 11D1BC7.14 generated in Chris Doe Lab<br>Chicken Anti-GFP: Aves Labs, GFP-1020<br>Rhodamin phalloidin: ThermoFisher Scientific, R415<br>Rabbit Anti-Zld: Purified in Melissa Harrison Lab<br>Rabbit Anti-GFP: Abcam, ab290<br>Rat Anti-Wor, clone CD#72B7AF3<br>Alexa Fluor® 647 AffiniPure Goat Anti-Rat IgG (H+L): Jackson Immunoresearch Cat#112-605-167<br>Alexa Fluor® 488 AffiniPure Donkey Anti-Chicken IgY (IgG) (H+L): Jackson Immunoresearch Cat#703-545-155<br>Goat anti-Rabbit IgG (H+L) Highly Cross-Adsorbed Secondary Antibody, Alexa Fluor 488: ThermoFisher Scientific Cat#A11034
Validation	Rabbit Anti-Ase: Validated in Weng et al., 2010<br>Rat Anti-Dpn: Validated in Lee et al., 2006b<br>Chicken Anti-GFP: Validation for immunohistochemistry on manufacturers website and in Komori H., 2018 and Rives-Quinto N., 2020<br>Rhodamin phalloidin: Validation for immunohistochemistry on manufacturers website.<br>Rabbit Anti-Zld: Validated in Harrison et al., 2010<br>Rabbit Anti-GFP: Validation for immunohistochemistry and immunoprecipitation on manufacturers website.<br>Rat Anti-Wor: Validated in Lee et al., 2006b

Animals and other organisms

Policy information about studies involving animals; ARRIVE guidelines recommended for reporting animal research

Laboratory animals	The following Drosophila melanogaster strains were used in this study. Sex was not considered and experiments were done at the third instar larval stage unless otherwise noted:

brat11/cyo,ActGFP (Lee et al., 2006a)
bratDf(2L)Exel8040/cyo,ActGFP (Bloomington Stocks 7847)
sfGFP-Zld (Hamm et al., 2017)
Six4::GFP (Bloomington Stocks 67733)
FlyLight GMR31F04-Gal4 (Bloomington Stocks 46187)
FlyLight GMR31D09-Gal4 (Bloomington Stocks 49676)
UAS-Zld (This study)
UAS-ZldZnF5 (This study)
UAS-ZldZnF3-6 (This study)
Worniu-Gal4, Ase-Gal80 (Neumuller et al., 2011)
Worniu-Gal4, Tub-Gal80ts (Lee et al., 2006a)
Erm(II)-Gal4 (Xiao et al., 2012b)
Erm(III)-Gal4 (Pfeiffer et al., 2008)
Opa-Gal4 (Bloomington Stocks 46979)
WT dpn-GFP::luciferase (This study)
Su(H) mut dpn-GFP::luciferase (This study)-also used at embryonic stage
Zld mut dpn-GFP::luciferase (This study)
Su(H)+Zld mut dpn-GFP::lucifearse (This study)-also used at embryonic stage
dpn1 (Bier et al., 1992)
hamDf(2L)Exel7071 (Bloomington Stocks 7843)
erml(2)5138 (Weng et al., 2010)
zld294 (Liang et al., 2008)
Notch RNAi (Bloomington Stocks 33611)
Tll-GFP 4912-+232 (This study)
Tll-GFP 4827-1114 (This study)
Tll-GFP 4827-2188 (This study)
Tll-GFP 4827-3158 (This study)
Tll-GFP 3158-1114 (This study)
Tll-GFP 2618-2040 (This study)

Wild animals: This study did not involve wild animals.

Field-collected samples: This study did not involve samples collected from the field.

Ethics oversight: No ethical approval or guidance was required for the use of D. melanogaster.

Note that full information on the approval of the study protocol must also be provided in the manuscript.

ChIP-seq

Data deposition

☒ Confirm that both raw and final processed data have been deposited in a public database such as GEO.

☒ Confirm that you have deposited or provided access to graph files (e.g. BED files) for the called peaks.

Data access links
May remain private before publication.
Sequencing data have been deposited in GEO under accession code GSE150931.

Files in database submission
GSM4561504 Zld_aZld_input_1
GSM4561505 Zld_aZld_ChIP_1
GSM4561506 Zld_aZld_input_2
GSM4561507 Zld_aZld_ChIP_2
GSM4561508 sfGFP-Zld_aGFP_input_1
GSM4561509 sfGFP-Zld_aGFP_ChIP_1

Genome browser session
(e.g. UCSC)
https://genome.ucsc.edu/s/elarson425/merged%20bw%20and%20peaks

Methodology

Replicates: Zld ChIP-seq experiments were generated from two biological replicates and validated with one replicate using an orthogonal antibody (GFP). Pearson correlation coefficiency can be found in Supplemental Figure 3B.

Sequencing depth: All sequencing reads were 50bp single-end sequencing.

Zld_aZld_ChIP_1
total reads:42790523
reads post-trim: 40950724
reads that align once: 27887810

Zld_aZld_input_1
total reads:96890763
reads post-trim: 94578177
reads that align once: 64432588

Zld_aZld_ChIP_2
total reads: 41614045
reads post-trim: 40523197
reads that align once: 28545728

Zld_aZld_input_2
total reads:77462355
reads post-trim: 76118920
reads that align once:55134801

sfGFP-Zld_aGFP_ChIP_1
total reads:88214348
reads post-trim: 85520933
reads that align once: 49406735

sfGFP-Zld_aGFP_input_1
total reads:79886931
reads post-trim: 77970231
reads that align once: 52280160

Antibodies

Rabbit Anti-Zld (Harrison et al., 2010)
Rabbit Anti-GFP: Abcam, ab290

Peak calling parameters

MACS version 2 was used with default parameters to identify bound regions of chromatin in samples (IP vs INPUT) for both replicates of Zld antibody ChIP in neuroblasts. The GFP antibody ChIP in the neuroblasts was called with the parameters above with the exception of lowering the m-fold value to -m 3 50 due to low IP efficiency. Peak summits were extended by 100bps on either side. High confidence Zld-bound regions in the neuroblasts were called as 200bp peak regions with 50% overlap in both replicates using the BEDtools intersect function. Regions belonging to contigs and unmapped chromosomes were removed. High-confidence regions used for analysis with the GFP antibody ChIP in neuroblasts were called as being bound with 50% overlap in both Zld antibody replicates and the GFP antibody replicate.

Data quality

Peaks were called with the default q-value (minimum FDR) cutoff of 0.01.

Software

Read quality was checked using FASTQC (version 0.11.9). Adapters and low-quality bases were removed using Trimmomatic-0.39. Reads were mapped to the dm6 genome assembly using Bowtie 2. Throughout SAMtools was used to filter and convert file formats. MACS version 2 was used with default parameters described above to call peaks. Visualization of genomic data was achieved by generation of z score-normalized bigWig files from merged read coverage of replicates and displayed using Gviz and the UCSC Genome Browser. Heatmaps were generated using deepTools2 with z score-normalized bigWig files. Average signal line plots were generated using SeqPlots from z score-normalized bigWig files at 10 base-pair resolution. Genomic annotations were performed with the Bioconductor R package ChIPseeker (Bioconductor version 3.9, ChIPseeker version 1.18.0) using default settings and the BDGP dm6 genome through TxDb.Dmelanogaster.UCSC.dm6.ensGene package (BDGP version 1.4.1, TxDb version 3.4.4). TSS regions were redefined as -500bps to +150bps. Peak to nearest gene assignments were done using default settings of the annotatePeak() function. To test for enrichment of motifs, de novo motif searches were done using the MEME-suite (version 5.1.1) and Hypergeometric Optimization of Motif Enrichment (HOMER, version 4.11). Additional motif searches were done using the Biostrings package in R (version 2.50.2). The vcountPattern() function was used to tally the number of regions containing at least one occurrence of a motif and the vmatchPattern() was used to locate regions containing a motif.
