# Supplementary material for: Cell-type-specific chromatin occupancy by the pioneer factor Zelda drives key developmental transitions in Drosophila
Source: Nat Commun. 2021 Dec 9;12:7153. doi: 10.1038/s41467-021-27506-y (PMC8660810; doi:10.1038/s41467-021-27506-y)
Supplement: Supplementary file 2 — Peer Review File [file 41467_2021_27506_MOESM2_ESM.pdf]

nature portfolio

Peer Review File

Cell-type-specific chromatin occupancy by the pioneer factor Zelda drives key developmental transitions in Drosophila

Open Access This file is licensed under a Creative Commons Attribution 4.0 International License, which permits use, sharing, adaptation, distribution and reproduction in any medium or format, as long as you give appropriate credit to the original author(s) and the source, provide a link to the Creative Commons license, and indicate if changes were made. In the cases where the authors are anonymous, such as is the case for the reports of anonymous peer reviewers, author attribution should be to 'Anonymous Referee' followed by a clear attribution to the source work. The images or other third party material in this file are included in the article's Creative Commons license, unless indicated otherwise in a credit line to the material. If material is not included in the article's Creative Commons license and your intended use is not permitted by statutory regulation or exceeds the permitted use, you will need to obtain permission directly from the copyright holder. To view a copy of this license, visit http://creativecommons.org/licenses/by/4.0/.

REVIEWER COMMENTS

Reviewer #1 (Remarks to the Author):

Larson et al. present a compelling study which defines a novel role for Zelda outside of its known function during zygotic genome activation. The authors use diverse approaches to identify the function of Zelda in type II neuroblasts.

The use of genomic approaches to map Zelda occupancy and accessibility in type II neuroblasts provides a new view of pioneer factor function.

However, functional studies which link the genomic occupancy of Zelda at important neuroblast regulators such as tll and dpn to occupancy and chromatin accessibility are necessary to support the conclusion that Zelda "defines enhancers" that regulate neuroblast function.

Major points:

It is difficult to evaluate the tll upstream region reporter fusions because the only nonfunctional region tested is in the reverse orientation. Because many factors with motifs involved in 3D organization which have been identified by the authors have a known dependence on orientation, it is important to have an additional reporter which is in the correct orientation. The authors could also mutate the noncanonical motif or make a truncation which removes just the putative Zelda bound enhancer.

In order to support the claim that Zelda defines enhancers in neuroblasts, the function of the enhancer should be tested in the absence or with overexpression of Zelda. This type of functional test is key to determine whether Zelda is acting directly at its binding site or whether it is bound because it identifies regions of open chromatin. The ATAC-seq data are consistent with a model in which Zelda identifies its binding site because chromatin is open at this site.

It would be helpful to identify motifs for other factors at the tll enhancer region to determine whether Zelda is acting alone or in combination with other factors at this site.

The authors have done a very nice functional study of the dpn regulatory region. It would be helpful to show ATAC-seq data at this locus to support the claim that
Zelda is defining enhancers.

Minor point:

Figure. 6A Venn diagram has numbers in wrong location.

Reviewer #2 (Remarks to the Author):

Zld is a well-characterized pioneer transcription factor involved in early Drosophila embryogenesis, with a key role in the zygotic genome activation. In the present study, the authors focused on a later stage in development, the molecularly defined neuroblasts (NB), and analyzed the functions of Zld during the undifferentiated state, through differentiation to the intermediate neural progenitor (INP), and, finally, during INP to NB reprogramming. These new experimental systems allow the authors to address two important questions in the field: #1) context-dependent Zld's functions and #2) the

molecular mechanisms to limit Zld's function for cell-fate commitment, which, on a broader scale, could explain the molecular obstacle to cellular reprogramming. Hence, this study potentially provides significant conceptual advances, particularly in an in vivo experimental model. Intriguingly, the authors found that Zld genomic occupancy was dramatically reorganized in NB and that Zld's action was limited by the transcriptional repressors, Erm and Ham. However, the results presented in the manuscript are mostly descriptive and fall short in providing the mechanistic insights behind these findings. For example, the readout of the loss/gain-of-function studies were endpoint phenotype, but not changes in chromatin state or in the binding of Zld and the repressors. Also, the correlational study between Zld's binding and chromatin openness does not prove causality. I believe this study would strongly benefit from more rigorous bioinformatic analysis (for point #1) and/or a more direct assessment of the molecular mechanisms (for point #2). The authors should be more circumspect in how they state the conclusions. Additional comments are listed below.

Additional comments:
• Given that Zld protein with mutations in zinc finger DNA binding domains promote the undifferentiated state in NB (Figure 1d) and that majority of NB-specific Zld binding sites do not contain Zld binding motif (Figure 4), Zld could bind to the chromatin through a protein-protein interaction with other TFs. This would be a very intriguing distinction from Zld binding in early embryos. More rigorous motif analyses could provide potential candidates of the binding partner of Zld in NB.
• The correlation between Zld binding and open chromatin in Figure 5 does not indicate there is a directional causality.
• Although the authors concluded that changes in the chromatin state mediated by Erm and Ham limit Zld's reprogramming capacity, no direct evidence was presented in the manuscript. For example, how do Erm and Ham change the chromatin state, and specifically at the Zld binding sites?

Reviewer #3 (Remarks to the Author):

This study addresses the role of the Zelda (Zld) transcription factor during Drosophila melanogaster larval brain development, with particular emphasis on the development of one particular type of neural progenitor cell type: the Type II neuroblasts (NB). Previous studies (PMID 29191977) have found that zld is a target of brat, and that zld gates the transition from NB identity to intermediate neural progenitor identity (INP). In this study, the authors build on these previous findings and attempt to shed further light upon the role of zld. They find evidence that zld acts with Notch to maintain Type II NBs in an undifferentiated state, and that Zld genomic occupancy in NBs is different from that in the embryo. These findings are of interest for a more specialized audience, but there are several reasons why I do not think the current journal is a good fit for this study.
First, I have several concerns regarding the validity of the findings. These pertain to my concerns regarding exclusively using brat mutants for the Zld ChIP-seq analysis (point 10). In addition, I am concerned over the interpretations of the data, because there is a lack of clarity regarding when the different genetic experiments are conducted/triggered, when assays are conducted, coupled with an apparent under-appreciation of the embryonic origin of all central brain NBs, including Type II NB, and the fact that Notch, dpn, erm, grh, all play roles during embryonic NB development. In simple terms: are the effect they see larval effects, or embryonic effects scored in the larvae?
Second, I am not convinced that, even if correct, the findings presented herein are sufficiently novel to justify publication in a journal with a broad readership (see e.g. point 12). This concern pertains both to the lack of an apparent conceptual advance and to an apparent lack of potential transference to other model systems. Specifically, zld appears to intersect with the highly evolutionarily Notch pathway. However, Zld has no clear orthologues in mammals, making it difficult to place these findings in the context of mammalian neurogenesis. Presumably, possibly, what zld does in flies is being done by some other gene(s) in mammals, but we do not know which gene, or even if this biology is conserved.

1) First, to set the stage for the comments below, all Drosophila central brain NBs are generated during embryogenesis. This also includes the Type II NBs. After a phase of neurogenesis, the majority of NBs enter quiescence, to enter into neurogenesis again during larval stages. Hence, the larval GOF and LOF genetic manipulations, which if I understand correctly are conducted during larval stages (again, the details of the experiments are not clear), are conducted upon an already established Type I and Type II landscape, and a mixed landscape of quiescent or active NBs (again, depending upon when the authors actually are inducing GOF and LOF, and when they analyse).

2) Figure 1b: How is Notch[RNAi] achieved? Gal4/UAS-RNAi, shRNA injection? And when is Notch knocked-down?

3) Figure 1c: When is Zelda ectopically expressed?

4) Figure 1c: The statement that Zelda generated more Type II NBs is based upon more Dpn cells, that also lack Ase. It would be reassuring to see some actual Type II marker being turned on.

5) Figure 1c: wor-Gal4 drives UAS expression in all NBs, but drives little, if any expression elsewhere. This indicates that zelda misexpression converts Type I NBs to Type II, as opposed to generating supernumerary Type II NBs by reprogramming other cells. Moreover, depending upon how much the lack of Ase expression is really a marker of Type II, one could argue that Zelda misexpression in Type I NBs merely drives maintained Dpn expression, but does not actually convert Type I to Type II.

6) Rows 148-149: This seems like an overstatement, given the comments above, and given that genetic interactions with Notch are observed for a vast number of genes.

7) Figure 2b: The wor-Gal4, Ase-Gal-80 driver combination would drive UAS-zld already in the embryo. When are the Type II NBs scored? In L3?

8) Figure 2c: What does the expression of dpn-GFP:luciferase look like if the authors show a whole brain lobe?

9) The manuscript contains a separate Discussion section, and yet the Results are riddled with over-the-top interpretations of the data. These statements should be moved to the Discussion.

10) Conducting ChIP-seq in NBs exclusively from brat mutants may lead to erroneous results. This is particularly concerning because the previous study of brat in Type II NBs revealed that brat binds to the zld and dpn RNAs and mediates their degradation. Hence, brat mutants have elevated and aberrant Zld and Dpn expression (PMID 29191977). Thus, the differences in the Zld binding in the embryo versus larval Type II NBs may not be connected to cell/tissue type, but rather WT versus brat mutant i.e., a Zld over-misexpression scenario.

11) Rows 233-239: The authors state that "Identified Zld-binding sites were located in promoters and enhancers and were enriched for the known Zld-binding motif, CAGGTA (Fig3b,c)." But the Zld sites only constitute a couple of percent of all sites, with the vast majority being so-called "GAF, CLAMP" sites. What these other sites represent is explained more under Figure 4, but the speculation that Zld ChIP-seq would bind "genomic features" in Type II NBs sounds speculative.

12) Figure 5: The notion that any TF-binding, as scored by ChIP-seq, correlates with open chromatin, as scored by ATAC-seq, is obvious, and has been observed in numerous publications. TFs generally do not bind closed chromatin, and hence these two assays strongly correlate. Similarly, Figure 6b: the notion that TF-binding and open chromatin also overlaps with regulatory regions, as assayed by reporter transgenes, is obvious, and has been observed in numerous publications. For instance, the Encode and modEncode projects have extensively demonstrated the connection between TF-binding,

ATAC-seq and regulatory regions.

13) Figure 6b: They should show the expression pattern of the reporter transgenes.

We appreciate that the reviewer's felt this was a "compelling study" that addressed "important questions." We have worked to address all of the concerns raised by the reviewers as detailed below. Briefly, we have:

1. Generated and analyzed additional transgenes to further define the neuroblast-specific *tll* enhancer and tested the requirement for Erm and Zelda in expression of reporters driven by this enhancer. Together these data further support our identification of a Zelda-bound, neuroblast-specific *tll* enhancer.
2. Included additional analysis of our developmental ATAC-seq time course, which suggests that Zelda is important for chromatin accessibility at sites containing its canonical binding motif (CAGGTA).
3. Provided additional evidence supporting the biological relevance of the system used, including quantitation of Zelda levels in neuroblasts of *brat* mutant brains, FISH experiments verifying the type II neuroblast identity of supernumerary neuroblasts, better explanations of the experimental design demonstrating the larval origin of the supernumerary neuroblasts.
4. Revised the text to be more circumspect in our conclusions.

REVIEWER COMMENTS

Reviewer #1 (Remarks to the Author):

Larson et al. present a compelling study which defines a novel role for Zelda outside of its known function during zygotic genome activation. The authors use diverse approaches to identify the function of Zelda in type II neuroblasts.

The use of genomic approaches to map Zelda occupancy and accessibility in type II neuroblasts provides a new view of pioneer factor function.

However, functional studies which link the genomic occupancy of Zelda at important neuroblast regulators such as tll and dpn to occupancy and chromatin accessibility are necessary to support the conclusion that Zelda "defines enhancers" that regulate neuroblast function.

Major points:

It is difficult to evaluate the tll upstream region reporter fusions because the only nonfunctional region tested is in the reverse orientation. Because many factors with motifs involved in 3D organization which have been identified by the authors have a known dependence on orientation, it is important to have an additional reporter which is in the correct orientation. The authors could also mutate the noncanonical motif or make a truncation which removes just the putative Zelda bound enhancer.

We thank the reviewer for these suggestions and agree that the recommended reporters could further test our conclusions of the requirement of the neuroblast-specific, Zelda-bound enhancer upstream of *tll*. Because neuroblast-specific, Zelda-binding sites do not contain a canonical Zelda motif, and our analysis has not identified

a likely Zelda-binding motif in these loci, we were unable to mutate a motif in the neuroblast-specific enhancer. Instead, we have taken the alternate suggestion from the reviewer and created two additional reporters: one containing only the embryo-specific Zelda-bound enhancer and one truncated version lacking the neuroblast-specific Zelda-bound enhancer (lines 406-411). They are both in the correct orientation (Fig. 6b, Supplementary Fig. 6b-c). We show that the reporter containing only the embryo-specific enhancer is not expressed in type II neuroblasts and that the larger region containing this enhancer and additional regulatory information is expressed at extremely low levels in type II neuroblasts. Together these additional reporters further strengthen our identification of a type II neuroblast-specific *tll* reporter that is bound by Zelda specifically in the neuroblasts.

In order to support the claim that Zelda defines enhancers in neuroblasts, the function of the enhancer should be tested in the absence or with overexpression of Zelda. This type of functional test is key to determine whether Zelda is acting directly at its binding site or whether it is bound because it identifies regions of open chromatin. The ATAC-seq data are consistent with a model in which Zelda identifies its binding site because chromatin is open at this site.

While our data support a role for Zelda at this type II neuroblast-specific enhancer, we do not think it is the only factor required. This is because while Tailless is necessary for type II neuroblast maintenance, Zelda is dispensable. (Fig. 1b). Thus, other factors, such as Btd, PntP1 and Tll itself, are likely capable of driving expression in the absence of Zelda. In retrospect, we understand that using the word “define” was misleading and have changed the wording throughout the manuscript to reflect the fact that Zelda binding is instrumental in identifying type II neuroblast-specific enhancers.

Upon the reviewer’s suggestion, we analyzed expression of GFP driven by the neuroblast-specific *tll* enhancer when *zelda* was depleted using RNAi. We did not identify any significant change in GFP levels (see below). The extensive data contained in the manuscript continue to support a functional role for Zelda in type II neuroblast fate, and our identification of this type II neuroblast-specific *tll* enhancer. Additionally, our analysis of the ATAC-seq time course continue to support a pioneering role for Zelda at a subset of regions.

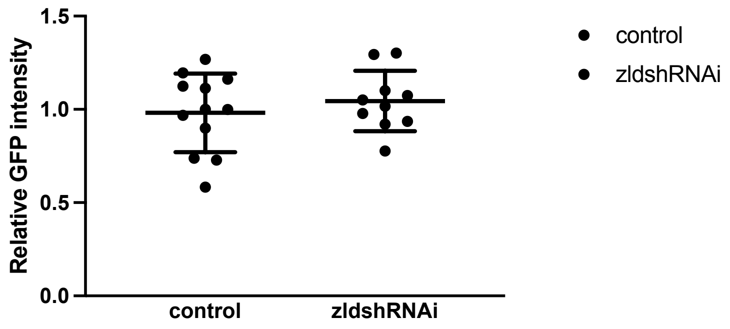

It would be helpful to identify motifs for other factors at the tll enhancer region to determine whether Zelda is acting alone or in combination with other factors at this site.

We share the reviewer's desire to identify motifs of possible cofactors that function with Zelda at loci similar to the *tll* enhancer region. Because our model suggests that Erm and Ham bind and decommission this enhancer, we initially identified potential Erm-binding sites based on our previous studies characterizing the sequence bound by Erm (Janssens et al. *Dev Cell* 2017) and now include asterisks in Fig. 6c marking the location of these Erm-binding motifs in the *tll* regulatory region. We have similarly identified Erm-binding motifs in the regulatory region of *Six4* (Supplementary Fig. 7b). In both cases, as predicted, Erm motifs are identified in the Zelda-bound, neuroblast-specific enhancers.

Despite extensive efforts by the Harrison lab (and likely others), there are no known Zelda cofactors. The Harrison lab has performed immunoprecipitation coupled with mass spectrometry, yeast two-hybrids, and BioID experiments to identify proteins that directly interact with Zelda. Although multiple proteins were identified as potential Zelda cofactors, we were unable to verify any of these through co-immunoprecipitation followed by immunoblot. We speculate that Zelda may interact transiently with a large number of factors leaving us unable to identify any direct protein interactors. This is supported by published literature that shows that Zelda localizes to transient hubs in the early embryo and that these hubs are important for chromatin binding of additional transcription factors Dorsal and Bicoid (Mir et al. *eLife* 2018, Dufourt et al. *Nat. Commun.* 2018, Yamada et al. *Current Bio* 2019).

Given this lack of known Zelda cofactors, we performed extensive *de novo* motif analyses at all classes of Zelda-bound regions in the neuroblasts to try to identify candidates. As outlined in the manuscript, the canonical Zelda-binding motif is enriched at regions that are bound by Zelda in both the embryo and neuroblasts, but it is not enriched at regions that are specifically bound by Zelda in neuroblasts. Rather, motifs of known promoter-binding factors, such as M1BP, DREF/BEAF-32 and GAF/CLAMP, are enriched. When we exclude promoters from this search, the motifs of these factors are no longer enriched. Thus, we propose that Zelda is being preferentially recruited to promoter regions that are known to open and accessible, possibly by one or more of these factors. Recently, the Harrison lab has shown that Zelda does not depend on GAF for chromatin occupancy in the early embryo (Gaskill et al. *eLife* 2021), but we cannot rule out a role for the other factors. The lab of Erica Larschan has recently investigated the role of CLAMP in determining Zelda binding in the early embryo and shown that it may promote Zelda binding at a subset of embryonic promoters (Duan et al. *eLife* 2021). We have now referred to this new finding in the Discussion (lines 577-579).

In addition to promoters, we also identified an enrichment for Zelda binding at enhancers in the neuroblasts. However, *de novo* motif searches limited to these regions

failed to identify enriched motifs that implicated additional cofactors. To further refine our analysis, we identified motifs enriched in only those enhancers that decreased in accessibility over differentiation. These searches also failed to identify clear motifs enriched at these sites when compared to regions that remained accessible (see provided heatmap at the end of the response to reviewers). The lack of enrichment of known motifs supports our model that rather than a factor or factors driving Zelda binding in the neuroblasts, chromatin architecture and accessibility have a strong influence on where Zelda binds.

The authors have done a very nice functional study of the dpn regulatory region. It would be helpful to show ATAC-seq data at this locus to support the claim that Zelda is defining enhancers.

Genome browser tracks of Zelda ChIP-seq and ATAC-seq in the neuroblasts are provided in Supplementary Fig. 5b. Zelda bound regions at the *dpn* locus are accessible in neuroblasts. Additionally, we have now included further analysis of the ATAC-seq data done during neuroblast differentiation (lines 440-459) and revised our wording to reflect that Zelda is pioneering at a subset of tissue-specific enhancers.

Minor point:

Figure. 6A Venn diagram has numbers in wrong location.

We thank the reviewer for catching this mistake. It has been fixed.

Reviewer #2 (Remarks to the Author):

Zelda is a well-characterized pioneer transcription factor involved in early Drosophila embryogenesis, with a key role in the zygotic genome activation. In the present study, the authors focused on a later stage in development, the molecularly defined neuroblasts (NB), and analyzed the functions of Zelda during the undifferentiated state, through differentiation to the intermediate neural progenitor (INP), and, finally, during INP to NB reprogramming. These new experimental systems allow the authors to address two important questions in the field: #1) context-dependent Zelda's functions and #2) the molecular mechanisms to limit Zelda's function for cell-fate commitment, which, on a broader scale, could explain the molecular obstacle to cellular reprogramming. Hence, this study potentially provides significant conceptual advances, particularly in an in vivo experimental model. Intriguingly, the authors found that Zelda genomic occupancy was dramatically reorganized in NB and that Zelda's action was limited by the transcriptional repressors, Erm and Ham. However, the results presented in the manuscript are mostly descriptive and fall short in providing the mechanistic insights behind these findings. For example, the readout of the loss/gain-of-function studies were endpoint phenotype, but not changes in chromatin state or in the binding of Zelda and the repressors. Also, the correlational study between Zelda's binding and

chromatin openness does not prove causality. I believe this study would strongly benefit from more rigorous bioinformatic analysis (for point #1) and/or a more direct assessment of the molecular mechanisms (for point #2). The authors should be more circumspect in how they state the conclusions. Additional comments are listed below.

We are pleased that the reviewer finds this study a conceptual advancement to the field. While we agree that more work is necessary to solidify the mechanistic details of our model, our ability to gain direct mechanistic insight into the differences between Zelda binding in the embryo and neuroblasts is limited by our biological system. However, we also believe that these studies represent an advancement in the pioneer factor field because they are performed in the context of a developmental system that enables phenotypic readouts in contrast to cell culture systems. Using currently available tools, we are limited in our ability to directly test the effect of Zelda binding on chromatin accessibility in the neuroblasts as that would require depleting Zelda from neuroblasts and performing ATAC-seq. However, prolonged *zelda* RNAi in a *brat* mutant brain causes a decrease in the number of type II neuroblasts (Reichardt et al. *EMBO Rep* 2017). Therefore, we would be unable to decipher whether any changes in accessibility were due to loss of Zelda or a change in cell type. Because of these limitations, we have edited the manuscript to be more circumspect in our conclusions.

To address our shared desire to provide more mechanistic insights, we have performed in-depth analysis of the ATAC-seq time course. Our findings from this analysis indicate that Zelda may be required to maintain accessibility at sites in the neuroblast where it is bound to its canonical motif. Specifically, Zelda-bound regions containing the canonical Zelda-binding motif (CAGGTA) rapidly lose accessibility as neuroblasts exit the stem-cell fate. By contrast, Zelda-bound regions that do not possess a CAGGTA motif lose accessibility more gradually over differentiation, supporting our model that the later expressed repressors Erm and Ham are necessary for silencing these enhancers. This analysis is now included in Fig. 6d and in Supplementary Fig. 5d,e (lines 440-459). This more extensive bioinformatic analysis has provided additional evidence for the role Zelda is playing in different classes of binding sites in the neuroblasts.

Additional comments:
• Given that Zelda protein with mutations in zinc finger DNA binding domains promote the undifferentiated state in NB (Figure 1d) and that majority of NB-specific Zelda binding sites do not contain Zelda binding motif (Figure 4), Zelda could bind to the chromatin through a protein-protein interaction with other TFs. This would be a very intriguing distinction from Zelda binding in early embryos. More rigorous motif analyses could provide potential candidates of the binding partner of Zelda in NB.

We agree with the reviewer that the distinction between Zelda-bound sites in the early embryo and larval neuroblasts is exciting, and we are eager to determine possible binding partners for Zelda in the neuroblast.

We performed extensive *de novo* motif analyses at all classes of Zelda-bound regions in the neuroblasts to try to identify candidates. As outlined in the manuscript, the canonical

Zelda-binding motif is enriched at regions that are bound by Zelda in both the embryo and neuroblasts, but it is not enriched at regions that are specifically bound by Zelda in neuroblasts. Rather, motifs of known promoter-binding factors, such as M1BP, DREF/BEAF-32 and GAF/CLAMP, are enriched. When we exclude promoters from this search, the motifs of these factors are no longer enriched. Thus, we propose that Zelda is being preferentially recruited to promoter regions that are known to be open and accessible, possibly by one or more of these factors. Recently, the Harrison lab has shown that Zelda does not depend on GAF for chromatin occupancy in the early embryo (Gaskill et al. *eLife* 2021), but we cannot rule out a role for the other factors. The lab of Erica Larschan has recently published a manuscript suggesting that CLAMP influences Zelda binding in the early embryo (Duan et al. *eLife* 2021). We have added a reference to this paper in the Discussion (lines 577-579).

In addition to promoters, we also identified an enrichment for Zelda binding at enhancers in the neuroblasts. However, *de novo* motif searches limited to these regions failed to identify enriched motifs that implicated additional cofactors. To further refine our analysis, we identified motifs enriched in only those enhancers that decreased in accessibility over differentiation. These searches also failed to identify clear motifs enriched at these sites when compared to regions that remained accessible (see provided heatmap at the end of the response to reviewers). The lack of enrichment of known motifs supports our model that rather than a factor or factors driving Zelda binding in the neuroblasts, chromatin architecture and accessibility have a strong influence on where Zelda binds.

Ongoing studies, beyond the scope of this manuscript, will combine biochemical and genomic strategies to try to identify mechanisms that may be driving this unique Zelda occupancy in the different tissues.

• The correlation between Zelda binding and open chromatin in Figure 5 does not indicate there is a directional causality.

We agree with this statement and apologize if the manuscript as previously written suggested otherwise. We have edited this section to better reflect this correlative relationship. Due to limitations of the biological system outlined above, we cannot directly test for causality. To provide more information on the relationship between Zelda expression and chromatin accessibility, we have performed additional in-depth analysis of the ATAC-seq done along the type II neuroblasts lineage during differentiation (lines 440-459).

To analyze the changes in chromatin accessibility as cells differentiate from a neuroblast to an intermediate progenitor, we performed k-means clustering on all regions that change in accessibility during our ATAC-seq time course, which reflected differentiation through the neuroblast lineage. We identified 6 clusters with distinct patterns of accessibility over the time course (Supplementary Fig. 5d). Zelda-bound sites in the neuroblasts were enriched in clusters 4, 5 and 6, which decrease in accessibility over the time course (29%) as compared to clusters 1, 2 and 3 that

increase in accessibility (15%). To identify factors that could be driving these changes in accessibility, we performed motif searches at the Zelda-bound sites in each of these clusters and identified that cluster 6 had the largest percentage of sites with the canonical Zelda motif (CAGGTA) (Supplementary Fig. 5e). This cluster is characterized by a rapid decrease in accessibility immediately following heat shock, correlating with cells exiting the neuroblast fate when Zelda levels drop. Based on this observation, we hypothesized that regions bound by Zelda in the neuroblasts that contain the canonical Zelda motif may be sites where Zelda is required for accessibility. We therefore specifically looked at changes in chromatin accessibility during differentiation at Zelda-bound sites that either had the canonical Zelda-binding motif (CAGGTA) or did not. We demonstrated that Zelda-bound regions with a canonical motif rapidly lost accessibility in the first 6 hours following heat shock, whereas those that lacked a canonical binding motif more gradually lost accessibility (Fig. 6d). Thus, both analysis of unbiased clustering as well as specific analysis of motif-containing sites demonstrated that Zelda binding to CAGGTA motifs is correlated with regions that rapidly lose accessibility as cells exit the neuroblast fate correlating with a dramatic reduction in Zelda levels. This suggests that at these sites Zelda may be responsible for promoting accessibility. By contrast, Zelda-bound regions that do not contain this motif lose accessibility less rapidly following stem-cell exit and may require additional factors for this loss in accessibility. Based on our data, we propose that some of these additional factors are Erm and Ham. We have previously shown that these two transcriptional repressors decommission *tll* expression during INP commitment through interactions with histone deacetylase 3 (Rives-Quinto et al. *eLife* 2020). Therefore, we suggest that their activity leads to decreased chromatin accessibility at those Zelda-bound enhancers that lose accessibility as cells transition from immature to mature INPs. We have further tested these interactions as described in response to the comment below.

• Although the authors concluded that changes in the chromatin state mediated by Erm and Ham limit Zelda's reprogramming capacity, no direct evidence was presented in the manuscript. For example, how do Erm and Ham change the chromatin state, and specifically at the Zelda binding sites?

Much of this evidence is contained in recent publications from the Lee and Harrison labs and others (Rives-Quinto et al. *eLife* 2020; Janssens et al. *Dev Cell* 2017). These papers have demonstrated that Erm functions to restrict the developmental potential of INPs. The histone deacetylase Hdac1/Rpd3 keeps the *erm* immature INP enhancer inactive in neuroblasts. Following asymmetric division, repressor down regulation relieves Rpd3 repression of *erm*. Once active, Erm represses genes that encode neuroblast-transcriptional activators. Ham is a negative regulator of type II neuroblast fate and is expressed after Erm in immature INPs. During INP commitment, Erm and Ham both function through histone deacetylation to continually inactivate transcription of the self-renewal factor Tll.

Genetic data presented in this manuscript (Fig. 6e) demonstrate that both Erm and Ham antagonize Zelda-mediated induction of the neuroblast fate. Furthermore, we identify Erm-binding motifs in the *tll* and *Six4* enhancers (Fig. 6c, Supplementary Fig. 7b). To

further test the role of Erm and Ham-mediated silencing, we analyzed GFP expression driven by the neuroblast-specific *tll* enhancer in larva heterozygous for null mutants in both *erm* and *ham* (lines 480-484). GFP expression is increased in this double heterozygous background (Fig. 6f). These new data, together with previously published data, support a model in which the type II neuroblast-specific, Zelda-bound enhancers (*tll* and *Six4*) are progressively silenced by Erm and Ham to inhibit reactivation in the INP.

Reviewer #3 (Remarks to the Author):

This study addresses the role of the Zelda (Zelda) transcription factor during Drosophila melanogaster larval brain development, with particular emphasis on the development of one particular type of neural progenitor cell type: the Type II neuroblasts (NB). Previous studies (PMID 29191977) have found that Zelda is a target of brat, and that Zelda gates the transition from NB identity to intermediate neural progenitor identity (INP). In this study, the authors build on these previous findings and attempt to shed further light upon the role of Zelda. They find evidence that Zelda acts with Notch to maintain Type II NBs in an undifferentiated state, and that Zelda genomic occupancy in NBs is different from that in the embryo. These findings are of interest for a more specialized audience, but there are several reasons why I do not think the current journal is a good fit for this study.
First, I have several concerns regarding the validity of the findings. These pertain to my concerns regarding exclusively using brat mutants for the Zelda ChIP-seq analysis (point 10). In addition, I am concerned over the interpretations of the data, because there is a lack of clarity regarding when the different genetic experiments are conducted/triggered, when assays are conducted, coupled with an apparent under-appreciation of the embryonic origin of all central brain NBs, including Type II NB, and the fact that Notch, dpn, erm, grh, all play roles during embryonic NB development. In simple terms: are the effect they see larval effects, or embryonic effects scored in the larvae?
Second, I am not convinced that, even if correct, the findings presented herein are sufficiently novel to justify publication in a journal with a broad readership (see e.g. point 12). This concern pertains both to the lack of an apparent conceptual advance and to an apparent lack of potential transference to other model systems. Specifically, Zelda appears to intersect with the highly evolutionarily Notch pathway. However, Zelda has no clear orthologues in mammals, making it difficult to place these findings in the context of mammalian neurogenesis. Presumably, possibly, what Zelda does in flies is being done by some other gene(s) in mammals, but we do not know which gene, or even if this biology is conserved.

We apologize to the reviewer for the lack of clarity when describing different genetic backgrounds and when the experiments were performed. All of the concerns regarding specific drivers raised by this reviewer are ones we considered during our experimental design. We have now detailed these drivers below and within the manuscript and explained how we are confident in the larval effects we reported. Additionally, we have

now verified that Zelda levels within a single neuroblast are equivalent in wild-type and *brat* mutant brains (see response below for details).

As for the second concern, we would like to respectively point out that while not conserved at the sequence level, over a decade of work has shown that, similar to Zelda in *Drosophila*, other pioneer factors are essential for activating the zygotic genome in vertebrates. Zelda was the first identified master regulator of the maternal-to-zygotic transition (Liang et al. *Nature* 2008) and was fundamental to our understanding of this process in vertebrates. Since the original identification of Zelda, mechanistic studies have laid the groundwork for our understanding of this key developmental transition in all metazoans. The pioneering functions of Zelda are also well described and similar to mammalian pioneer factors (Schulz et al. *Genome Res* 2015, Sun et al. *Genome Res* 2015, McDaniel et al. *Mol Cell* 2019, Fernandez Garcia et al. *Mol Cell* 2019 and others). As such, studies of Zelda-mediated transcriptional networks in the *Drosophila* embryo have provided significant insights into our understanding of how pioneer factors drive changes in cell fate.

1) First, to set the stage for the comments below, all Drosophila central brain NBs are generated during embryogenesis. This also includes the Type II NBs. After a phase of neurogenesis, the majority of NBs enter quiescence, to enter into neurogenesis again during larval stages. Hence, the larval GOF and LOF genetic manipulations, which if I understand correctly are conducted during larval stages (again, the details of the experiments are not clear), are conducted upon an already established Type I and Type II landscape, and a mixed landscape of quiescent or active NBs (again, depending upon when the authors actually are inducing GOF and LOF, and when they analyse).

We apologize for any lack of clarity when discussing the drivers used in each experiment. All drivers used express in the type II neuroblast lineage and all dissections and imaging were done in the third instar larva. We have clarified exactly when these lines drive expression in the type II neuroblast lineage in the Methods section (lines 644-653). Expression patterns can be seen in Fig. 1a and are also listed here:

Wor-Gal4, Ase-Gal80 combines *Wor-Gal4* that expresses in type I and II neuroblasts with the Gal4 inhibitor Gal80 under the Ase promoter to obtain a line that drives expression specifically in type II neuroblasts (Nuemuller et al., 2011).

Wor-Gal4, Tub-Gal80ts is temperature sensitive thus upon temperature shift Gal80 repression on Gal4 is relieved and expression is driven. The temperature shift is done from 24 to 96 hours after egg laying (L1-L3 stages) and therefore is exclusive to the larval stages. These results specifically exclude the concerns regarding the embryonic neuroblasts.

Erm(II)-Gal4 drives expression in the Ase- immature INPs through Ase+ immature INPs and mature INPs.

Erm(III)-Gal4 drives expression in the Ase+ and mature INPs.

Opa-Gal4 drives expression in the mature INPs.

We agree with the reviewer that it is important to consider the origins of the neuroblast population. We carefully selected drivers that would eliminate any confusion about the origin of the end-point phenotypes reported. Specifically, using the combination of *Wor-Gal4, Tub-Gal80ts* we can limit transgene expression to a period of heat shock. Using this system, we drove expression of Zelda exclusively in the larva and observed the same supernumerary neuroblast phenotype as when expression was driven by *Wor-Gal4, Ase-Gal80* (Fig. 1c). These data allow us to be confident that the effects are caused by expression in the larval neuroblast and not the embryo.

2) Figure 1b: How is Notch[RNAi] achieved? Gal4/UAS-RNAi, shRNA injection? And when is Notch knocked-down?

Wor-Gal4 driven Notch RNAi (BDSC#33611) clones were induced by heat shock at 37°C for 90 minutes at 24 hours after larval hatching. Brains were dissected for clone analysis at 72 hours after clone induction (lines 670-674).

3) Figure 1c: When is Zelda ectopically expressed?

As labeled beneath the images, the *Wor-Gal4, Tub-Gal80ts* driver was used to overexpress Zelda in Fig. 1c. As stated in the text, embryos were cultured at a restrictive temperature to suppress *Zelda* transgene expression and larvae were cultured at a higher permissive temperature for 72 hours until dissection. As now clarified in the methods and figure legend, this expression is limited to the larval stages (L1-L3).

4) Figure 1c: The statement that Zelda generated more Type II NBs is based upon more Dpn cells, that also lack Ase. It would be reassuring to see some actual Type II marker being turned on.

We have now included Supplementary Fig. 1b where we used *in situ* hybridization for the type II neuroblast marker *Sp1*. The expression of *Sp1* in the supernumerary neuroblasts produced upon overexpression of Zelda confirms that they are type II neuroblasts (lines 133-135).

5) Figure 1c: wor-Gal4 drives UAS expression in all NBs, but drives little, if any expression elsewhere. This indicates that zelda misexpression converts Type I NBs to Type II, as opposed to generating supernumerary Type II NBs by reprogramming other cells. Moreover, depending upon how much the lack of Ase expression is really a marker of Type II, one could argue that Zelda misexpression in Type I NBs merely drives maintained Dpn expression, but does not actually convert Type I to Type II.

As stated in response to 4) above, we have confirmed using Sp1 transcription that these are supernumerary type II neuroblasts and not type I neuroblasts. In addition to

molecular markers, we took the location of supernumerary type II neuroblasts into consideration when determining where the supernumerary type II neuroblasts come from. All type II neuroblasts are located at the dorsal region or dorsoposterior medial region of the larval brain lobe (Boone & Doe *Dev Neurobiol* 2008). They don't exist in the ventral region of the brain lobe or ventral nerve code where type I neuroblasts exist. This segregation of location is useful in determining if a factor has the ability to convert type I neuroblasts into type II neuroblasts. For example, overexpression of *tll* by *Wor-Gal4* creates type II neuroblasts by conversion of type I neuroblasts in the ventral region of the brain lobe and ventral nerve cord (Rives-Quinto et al. *eLife* 2020; Hakes & Brand *eLife* 2020). The experiments in Fig.1c used the same experimental system to drive a *zelda* transgene. In this experiment, we found supernumerary type II neuroblasts in the dorsal region and dorsoposterior medial region of the brain lobe, but not in the ventral region of the brain lobe or ventral nerve cord. This phenotype is similar to that induced by overexpression of the constitutive active form of Notch that promotes the undifferentiated state of type II neuroblasts. These support our conclusion that overexpression of Zelda promotes the undifferentiated state of type II neuroblasts rather than overexpression of Zelda converting type I neuroblasts to type II neuroblasts.

Furthermore, in Fig. 2, we limited Gal4 activity in all type I neuroblasts by using *Ase-Gal80*. Since *Ase-Gal80* expresses specifically in type I neuroblasts and is able to inhibit Gal4 function in these cells, the combination of *Wor-Gal4* with *Ase-Gal80* allows us to drive UAS-transgene expression only in type II neuroblasts and their progeny cells. In Fig. 2a, we observed supernumerary type II neuroblasts in the dorsal region and dorsoposterior medial region of the brain lobe by using a combination of *Wor-Gal4* and *Ase-Gal80*, indicating that supernumerary type II neuroblasts originate from cells of type II neuroblast lineages. In fact, overexpression of Zelda in immature INPs by *erm-Gal4* (II) also drove supernumerary type II neuroblast phenotype (Fig. 2a).

6) Rows 148-149: This seems like an overstatement, given the comments above, and given that genetic interactions with Notch are observed for a vast number of genes.

We did not mean to imply that this interaction was unique to Zelda and merely conveyed the genetic interaction observed. We have revised the text (now lines 152-154).

7) Figure 2b: The wor-Gal4, Ase-Gal-80 driver combination would drive UAS-Zelda already in the embryo. When are the Type II NBs scored? In L3?

Yes, the type II neuroblasts were scored in L3. However, heat shock experiments in Fig. 1c show supernumerary neuroblasts are formed when expression is limited to the larva.

8) Figure 2c: What does the expression of dpn-GFP:luciferase look like if the authors show a whole brain lobe?

We have now included whole brain lobe images of the dpn-GFP:luciferase reporters (Supplementary Fig. 2c).

9) The manuscript contains a separate Discussion section, and yet the Results are riddled with over-the-top interpretations of the data. These statements should be moved to the Discussion.

We have worked to move these statements while retaining some explanation of the interpretation that allows the reader to understand the motivation for additional experiments.

10) Conducting ChIP-seq in NBs exclusively from brat mutants may lead to erroneous results. This is particularly concerning because the previous study of brat in Type II NBs revealed that brat binds to the Zelda and dpn RNAs and mediates their degradation. Hence, brat mutants have elevated and aberrant Zelda and Dpn expression (PMID 29191977). Thus, the differences in the Zelda binding in the embryo versus larval Type II NBs may not be connected to cell/tissue type, but rather WT versus brat mutant i.e., a Zelda over-misexpression scenario.

We thank the reviewer for their attention to these data. The paper mentioned shows that Brat degrades *zelda* mRNA and leads to reduced protein levels in the immature INP following asymmetric division and not in the neuroblast itself. However, to specifically address whether Zelda is being overexpressed in individual nuclei of a *brat* mutant brain we quantified levels of endogenously tagged GFP-Zelda expression in neuroblasts of a *brat* mutant brain as compared GFP-Zelda levels in neuroblasts of a wild-type background (lines 224-229). Relative GFP expression is the same in individual neuroblasts of wild type and *brat* mutants, indicating that Zelda protein levels are the same per neuroblast. These data are now included in Supplementary Fig. 3a. Thus, increased Zelda levels in the brain as a whole reflects the vast excess of type II neuroblasts rather than increased Zelda in the nuclei of individual cells.

11) Rows 233-239: The authors state that “Identified Zelda-binding sites were located in promoters and enhancers and were enriched for the known Zelda-binding motif, CAGGTA (Fig3b,c).” But the Zelda sites only constitute a couple of percent of all sites, with the vast majority being so-called “GAF, CLAMP” sites. What these other sites represent is explained more under Figure 4, but the speculation that Zelda ChIP-seq would bind “genomic features” in Type II NBs sounds speculative.

While the percentage of all Zelda-bound sites in the neuroblasts that contain a canonical Zelda motif is smaller than the percentage of sites containing a GAF/CLAMP motif, the Zelda motif is still significantly enriched at these sites. The enrichment of the Zelda motif at all Zelda-bound neuroblast sites is driven by the Zelda-binding sites that are shared with the early embryo, which only constitute about a third of total Zelda-binding sites in the neuroblasts. The majority of Zelda-bound sites in the neuroblasts are unique to that cell type and not enriched for the canonical Zelda motif.

Additionally, we show that Zelda binding is enriched at promoters in the neuroblasts compared to a random distribution of sites (Supplementary Fig. 4a). GAF/CLAMP and

the motifs they bind are known to be enriched at promoters so the high percentage of Zelda-bound sites containing this motif is unsurprising given the genomic distribution of sites.

12) Figure 5: The notion that any TF-binding, as scored by ChIP-seq, correlates with open chromatin, as scored by ATAC-seq, is obvious, and has been observed in numerous publications. TFs generally do not bind closed chromatin, and hence these two assays strongly correlate. Similarly, Figure 6b: the notion that TF-binding and open chromatin also overlaps with regulatory regions, as assayed by reporter transgenes, is obvious, and has been observed in numerous publications. For instance, the Encode and modEncode projects have extensively demonstrated the connection between TF-binding, ATAC-seq and regulatory regions.

While the reviewer is correct in that many TFs do not bind closed chromatin, Zelda is an extremely well-characterized pioneer factor, which are known for their role in binding closed chromatin and promoting accessibility. Our findings in this manuscript are distinct, in that we provide evidence that at the majority of bound sites, Zelda is not able to bind closed chromatin in the larval neuroblasts and this is contrary to what is observed in the early embryo. Examples of tissue-specific binding and function by pioneer factors in various model organisms are prevalent throughout the pioneer factor literature and are outlined in our recent review (Larson et al., Mol Cell, 2021). However, few of these factors have been studied outside of cell culture and within the context of multiple different tissues of a developing organism.

13) Figure 6b: They should show the expression pattern of the reporter transgenes.

The expression pattern of the reporter transgenes are included in Supplementary Fig. 6.

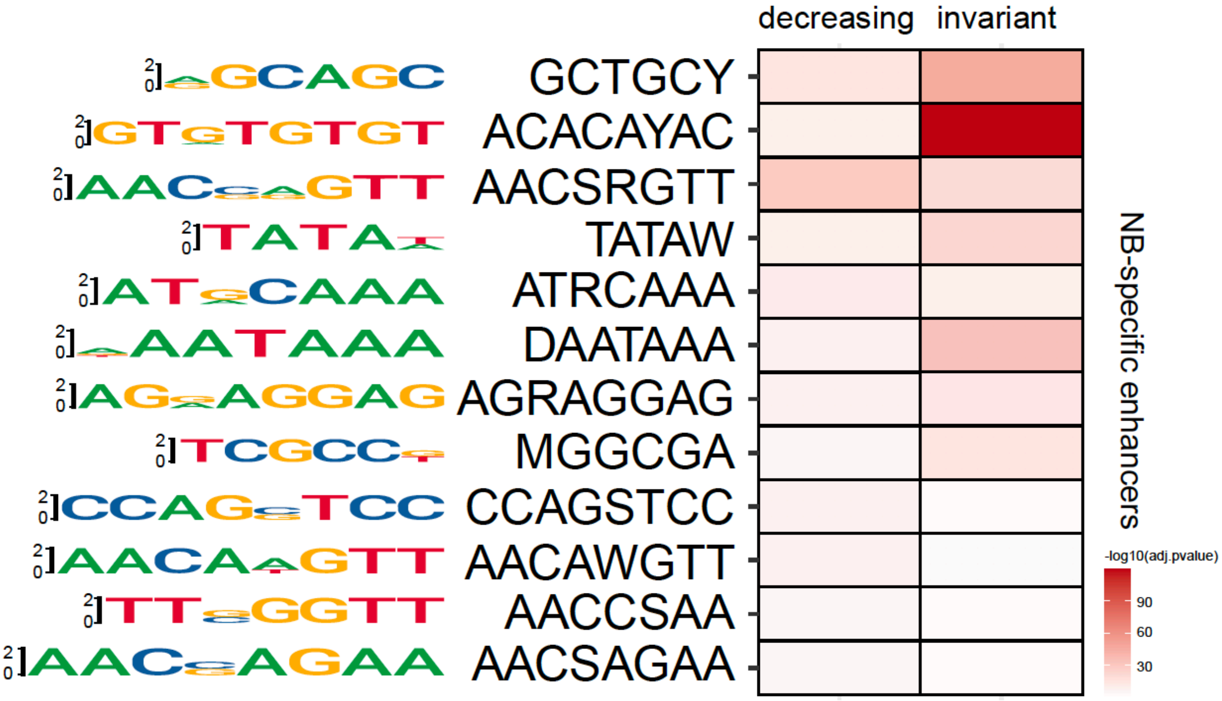

Limited motif enrichment at Zelda-bound, non-promoter regions. Enrichment in regions that either lose accessibility over the ATAC-seq time course (decreasing) or remain unchanged (invariant) of motifs enriched at regions that are Zelda-bound, lose accessibility and located in non-promoter regions (presumed enhancers). Color indicates significance value of various motifs compared to shuffled background. None of the motifs are significantly different between the two classes.

REVIEWERS' COMMENTS

Reviewer #1 (Remarks to the Author):

The authors addressed my key concerns through new experimentation and analysis. The main advance and important impact of this work is showing a role for pioneer factors beyond function in the early embryo even though all of the mechanisms have not been fully identified.

Reviewer #2 (Remarks to the Author):

The authors have satisfactorily addressed my comments with extensive additional experiments and analyses. In particular, their time-course ATAC analysis in the developmental system reinforced their model and provided the exciting distinction of potential Zelda's actions depending on the binding sites. This presents a conceptual advancement in the pioneer factor field. The conclusions in the revised manuscript reflect well the nature of their results. I now recommend the publication of this work in the journal.

Reviewer #3 (Remarks to the Author):

The authors have greatly improved the manuscript, by adding in description of stages, drivers, several control experiments and by tempering the language in places. However, three of my original points still concern me (below).

1) Thank you for clarifying the drivers and stages for heat-shock. However, Erm-Gal4 expression does not appear to be temperature-controlled by Gal80. Would it not therefore drive in INPs within the embryonic Type II NB lineages?

10) I am still concerned that all the Zelda ChIP-seq was conducted in brat mutants, and not on wild type. I understand that the limited number of Type II NBs present in the wt brain makes the wt analysis difficult, but it is nevertheless a concern.

12) If I understand correctly, in the neuroblast context, Zelda does not interact with DNA as a typical pioneer factor, binding even to closed chromatin, but rather behaves like a "typical" TF, and primarily binds to open chromatin? But this is not a surprising finding. Indeed, they state that "Examples of tissue-specific binding and function by pioneer factors in various model organisms are prevalent throughout the pioneer factor literature and are outlined in our recent review (Larson et al., Mol Cell, 2021)." They key thing to address now would be why pioneer factors sometimes loose their pioneer potency.

Response to Reviewers

Reviewer #1 (Remarks to the Author):

The authors addressed my key concerns through new experimentation and analysis. The main advance and important impact of this work is showing a role for pioneer factors beyond function in the early embryo even though all of the mechanisms have not been fully identified.

Reviewer #2 (Remarks to the Author):

The authors have satisfactorily addressed my comments with extensive additional experiments and analyses. In particular, their time-course ATAC analysis in the developmental system reinforced their model and provided the exciting distinction of potential Zelda's actions depending on the binding sites. This presents a conceptual advancement in the pioneer factor field. The conclusions in the revised manuscript reflect well the nature of their results. I now recommend the publication of this work in the journal.

We thank both Reviewers 1 and 2 for their prior suggestions that made this a stronger manuscript and are pleased that they recommend the manuscript for publication.

Reviewer #3 (Remarks to the Author):

The authors have greatly improved the manuscript, by adding in description of stages, drivers, several control experiments and by tempering the language in places. However, three of my original points still concern me (below).

1) Thank you for clarifying the drivers and stages for heat-shock. However, Erm-Gal4 expression does not appear to be temperature-controlled by Gal80. Would it not therefore drive in INPs within the embryonic Type II NB lineages?

The complete genotype of the driver is *Erm-Gal4,Tub-Gal80[ts]*. Gal80[ts] driven by the *tubulin* promoter is ubiquitously expressed in all cells in all stages and inhibits *Erm-Gal4* activity during embryogenesis. By shifting the temperature to 33ºC in larval stages, we inactivate Gal80[ts] allowing *Erm-Gal4* to become active only in larval development.

10) I am still concerned that all the Zelda ChIP-seq was conducted in brat mutants, and not on wild type. I understand that the limited number of Type II NBs present in the wt brain makes the wt analysis difficult, but it is nevertheless a concern.

We understand the concern. However, the fact that the *brat* mutant brains can differentiate along the lineage in a synchronous manner, that Zelda levels are similar in *brat* mutant and wild-type neuroblasts, and that the binding sites we identified in *dpn* are functionally relevant all support the use of the system.

12) If I understand correctly, in the neuroblast context, Zelda does not interact with DNA as a typical pioneer factor, binding even to closed chromatin, but rather behaves like a “typical” TF, and primarily binds to open chromatin? But this is not a surprising finding. Indeed, they state that “Examples of tissue-specific binding and function by pioneer factors in various model organisms are prevalent throughout the pioneer factor literature and are outlined in our recent review (Larson et al., Mol Cell, 2021).” They key thing to address now would be why pioneer factors sometimes lose their pioneer potency.

We absolutely agree that a key question moving forward, both for this work and for the pioneer factor field more generally, is what governs pioneer factor activity. This is interesting both from the perspective of what aspects of chromatin may limit pioneer-factor binding and how the presence of cofactors influences pioneer factors. Here we have importantly identified a system in which a pioneer factor shows different activity in discrete tissues of a developing organism, and, in the future, we will now use the embryo and neuroblast to address these important questions.
